# F-box protein FBXO30 mediates retinoic acid receptor γ ubiquitination and regulates BMP signaling in neural tube defects

**DOI:** 10.1038/s41419-019-1783-y

**Published:** 2019-07-18

**Authors:** Xiyue Cheng, Pei Pei, Juan Yu, Qin Zhang, Dan Li, Xiaolu Xie, Jianxin Wu, Shan Wang, Ting Zhang

**Affiliations:** 1 Beijing Municipal Key Laboratory of Child Development and Nutriomics, Capital Institute of Pediatrics, 100020 Beijing, China; 2 Graduate School of Peking Union Medical College, 100730 Beijing, China; 3 Department of Biochemistry and Molecular Biology, Shanxi Medical University, 030001 Taiyuan, Shanxi China; 4 Institute of Basic Medical Sciences, Chinese Academy of Medical Science, 100730 Beijing, China

**Keywords:** Developmental neurogenesis, Cellular neuroscience

## Abstract

Retinoic acid (RA), an active derivative of vitamin A, is critical for the neural system development. During the neural development, the RA/RA receptor (RAR) pathway suppresses BMP signaling-mediated proliferation and differentiation of neural progenitor cells. However, how the stability of RAR is regulated during neural system development and how BMP pathway genes expression in neural tissue from human fetuses affected with neural tube defects (NTDs) remain elusive. Here, we report that FBXO30 acts as an E3 ubiquitin ligase and targets RARγ for ubiquitination and proteasomal degradation. In this way, FBXO30 positively regulates BMP signaling in mammalian cells. Moreover, RA treatment leads to suppression of BMP signaling by reducing the level of FBXO30 in mammalian cells and in mouse embryos with NTDs. In samples from human NTDs with high levels of retinol, downregulation of BMP target genes was observed, along with aberrant FBXO30 levels. Collectively, our results demonstrate that RARγ levels are controlled by FBXO30-mediated ubiquitination and that FBXO30 is a key regulator of BMP signaling. Furthermore, we suggest a novel mechanism by which high-retinol levels affect the level of FBXO30, which antagonizes BMP signaling during early stage development.

## Introduction

The vertebrate neural tube is the precursor to the central nervous system (CNS). Neural tube defects (NTDs) are a group of birth defects caused by incomplete neural tube closure during embryonic development^1,2^. Failure to complete closure of the craniofacial region leads to exencephaly, and if the exposed brain tissue is degraded it appears as anencephaly, which usually causes embryonic lethality^3,4^. NTDs are caused by the external environment and genetic factors. Imbalance of nutrient intake (e.g., folate or vitamin A) during peripregnancy is an important risk factor^5^. When vitamin A enters the body, it is first oxidized to retinaldehyde and then further oxidized to retinol, i.e., retinoic acid (RA)^6–8^. The primary mechanism by which vitamin A regulates CNS development and physiological actions in the body, is through its oxidation to the active metabolite, RA^9^. Excessive intake of preformed vitamin A has been linked to teratogenicity in both human and animal’s studies^10,11^. During the process of neural tube closure, the distribution of RA in neuronal cells has stage- and tissue-specificity^12^. Vitamin A deficient quail embryos and mouse embryos completely lacking RA signaling exhibit hindbrain defects^13^. Excess RA causes many of the same embryonic developmental defects seen with vitamin A deficiency, which is why vitamin A intake levels should be monitored carefully during pregnancy^14^.

RA exerts its functions through RA receptors (RARs)^15^. RARs are members of the nuclear receptor superfamily^16^, and include RARα, RARβ, and RARγ, and the heterodimeric retinoid X receptor superfamily. RA synthesized in specific locations regulates transcription by interacting with nuclear RA receptors bound to RA response elements near target genes^17^. Disorders associated with RAR and its signaling pathway is closely related to neural development and neural tube defects^18–22^. During development of the nervous system, the RA signaling pathway interacts with fibroblast growth factor, WNT, and bone morphogenic protein (BMP) signaling pathways to coordinately regulate the development of the nervous system^23–26^.The RA/RAR pathway inhibits BMP signaling by mediating the ubiquitylation and degradation of phosphorylated Smad1; this antagonizes BMP-regulated proliferation and differentiation of neural progenitor cells^27^. While the existence of signaling pathways downstream of the RA receptors is well-known, it remains unclear precisely how RA signaling participates in neural tube closure at the cellular level. Given the significant role of RAR during embryonic development, the stability and activity of RARs needs to be tightly controlled and RARs are degradation by the ubiquitin-proteasome pathway.

It is currently unclear how RARs are negatively regulated. A recent study showed that MDM2 acts as a ubiquitin protein ligase of RARα, which can promote the polyubiquitylation and degradation of RARα^28^. To search for more RAR regulators, immunoprecipitation/mass spectrometry (IP/MS) using an RAR antibody was performed and a candidate interactor was the F-box protein, F-box domain-containing protein 30 (FBXO30). Many aspects of embryonic development are controlled by the ubiquitination process. An important class of ubiquitin E3 enzymes in this regard is SCF (Skp1-Cullin1-F-box)-type ligases, for which F-box proteins are substrates^29–33^. F-box proteins are associated with various biological processes, such as embryonic development, adult bone formation, and tumorigenesis. FBXW7 and FBXL1 are important molecules in tumor development^34,35^. A recent study showed that FBXL15 is involved in formation of the dorsal-ventral axis of zebrafish embryos, and that FBXO3 mediates the ubiquitination of Smurf1 and promotes BMP pathway activity^36^. Recently, the studies showed that FBXO38 is a specific E3 ubiquitin ligase of PD-1 and essential for maintaining the antitumor activity of T cells. However, the function and substrates of F-box-type proteins are mostly unclear. While there are over 60 human F-box proteins, nearly 70% of them are not well characterized, including FBXO30. The purpose of this study was to screen F-box family proteins for selective regulators of RARs involved in neural tube closure control.

Here, we report that FBXO30 can interact with RARγ and promote its ubiquitin-mediated degradation. Our study reveals that the ubiquitin protein ligase, FBXO30, negatively regulates the stability of RARγ protein, and functions to regulate BMP signaling. This finding was validated in RA-induced mouse NTD models and high-retinol level human fetuses NTD, indicating that abnormal expression of FBXO30 may participate in neural development and be a factor in the occurrence of neural tube defects.

## Materials and methods

### Plasmids and antibodies

Full-length FBXO30, zinc finger, and F-box mutants were cloned into Flag-CMV-2 vectors, full-length RARγ pCMV-Myc were cloned into as indicated. Antibodies used in immunoblotting: anti-Myc (Abcam), anti-flag-HRP (sigmar), FBXO30 antibody (Santa-Cruz), RARγ1 antibody (CST), GAPDH antibody (proteintech), H3-HRP antibody (abcam), and p-smad1/5 antibody (CST).

### Cell culture

HEK293 and NT2/D1 (NT2/D1 human embryonic teratocarcinoma) cell lines were obtained from National Infrastructure of Cell Line Resource. HEK293 were cultured in MEM (Hyclone) with 10% fetal bovine serum (Gibco). NT2/D1 were cultured in DMEM (Gibco) with 10% fetal bovine serum. The cells were maintained at 37 °C in a humidified atmosphere with 5% CO_2_.

### Mass spectrometry

Thermo-fisher Scientific EASY-nLC 1000 was used to separate peptides in UHPLC with a EASY-Spray column (12 cm × 75 μm, C18, 3 μm), accompanied with a precolumn (Acclaim Pepmap100 column, 2 cm × 100 μm, C18, 5 μm). Mobile phase A was 100% ultra-pure water with 0.1% formic acid. Mobile phase B was 100% acetonitrile with 0.1% formic acid. Flow rate was 350 nL/min. The separation gradient was running with solution B in the first 5 min, increasing to 15% solution B for the next 35 min, kept increasing to 25% solution B for another 25 min, then 35% solution B for 5 min, 95% solution B for the last 15 min. Eluent was detected in mass spectrophotometer (Thermo, Q-Exactive). Ion source was set to 2.1 kV. Spectrums were processed using Proteome Discoverer 2.1 software.

### Co-IP assay

Cells were harvested 48 h after transfection, transfection reagent for lipofetamine2000 (Invitrogen) and lysed in radioimmunoprecipitation assay lysis buffer (beyotime, P0013D) supplemented with protease inhibitor cocktail (Roche). Take 500 μL of freshly extracted whole protein lysate for co-IP, take 50 μL as input, add appropriate antibody (1–2 μg) to lysis, add 1–2 μg of Normal IgG from the same source, and mix and incubate at 4 °C for 5–6 h. Then, add protein A/G agarose(Santa) 50 μL/tube and incubate overnight (10–12 h) at 4 °C.The resulting immunoprecipitates were washed at least three times in HEPES lysis buffer (20 mM HEPES pH 7.2, 50 mM NaCl, 0.5% TritonX-100, 1 mM NaF, 1 mM dithiothreitol) before being resolved by sodium dodecyl sulfate polyacrylamide gel electrophoresis and immunoblotted with indicated antibodies.

### RNA interference

The FBXO30 siRNA and RARγ siRNA sequences of the strands were as follows:

FBXO30-Homo-1^#^, sense (5′-3′) GUAGCCACCAUGAUGUCAATT, antisense (5′-3′) UUGACAUCAUGGUGGCUACTT; FBXO30-Homo-2^#^, sense (5′-3′) GGCCCGAAAUAAAGUUGCUTT, antisense (5′-3′) AGCAACUUUAUUUCGGGCCTT; FBXO30-Homo-3^#^, sense (5′-3′) GGAGGAAAUAGGAGCAGUATT, antisense (5′-3′) UACUGCUCCUAUUUCCUCCTT; Negative control, sense (5′-3′) UUCUCCGAACGUGUCACGUTT, antisense (5′-3′) ACGUGACACGUUCGGAGAATT; RARγ-Homo, sense (5′-3′) GCCGAAGCAUCCAGAAGAATT, antisense (5′-3′) UUCUUCUGGAUGCUUCGGCTT. All siRNA transfections were performed using Lipofectamine 2000 (Invitrogen) according to the commercialized protocol, and the RNAi efficiency was assessed by western blotting analysis.

### Protein half-life assay

For RARγ-half-life assay, cells were treated with the protein synthesis inhibitor cycloheximide (Sigma, 50 μg/mL) for the indicated durations before harvest.

### Luciferase reporter assay

BRE-luciferase reporter assay was carried out as described previously^37^. Cells were lysed with Cell Lysis Buffer (Yuan Ping Hao),and luciferase activities in cell extracts were determined with a dual-luciferase assay system (Promega).

### Real-time polymerase chain reaction (PCR)

RNA was extracted from cell lines using TRIzol (Invitrogen) according to the manufacturer’s protocol. cDNA was synthesized using a 5× All-In-One RT Master Mix(with Accu RT Genomic DNA Removal Kit, abm). Real-time PCR assay was performed using a Quant Studio TM 7 Flex PCR System (Thermo). PCR reactions were carried out in 20 μL reactions using EvaGeen2× qPCRMasterMix-low ROX (Abm) and 0.2 μM specific primers. Primer sequences for specific genes are available upon request and listed in Supplementary Table S1.

### Fluorescence analysis

For detection of subcellular localization by immunofluorescence, after fixed with 4% paraformaldehyde and permeabilized in 0.1% Triton X-100 (phosphate-buffered saline (PBS)), cells were incubated with the indicated FBXO30 (dilution 1:100; Santa-Cruz) and RARγ1 antibodies (dilution 1:200; CST) or p-Smad1/5 antibody (dilution 1:200;CST)for overnight at 4 °C, followed by incubation with Alexa Fluor488-conjugated or Alexa Fluor594-conjugated secondary antibody (dilution 1:200;Zhongshanjinqiao) for 1 h at 37 °C. The nuclei were stained with DAPI (Solarbio), and images were visualized with a Zeiss LSM 510 Meta inverted confocal microscope.

### Animals

C57BL/6 mice, 8–10 weeks old, weighing 18–23 g, SPF grade, purchased from the Experimental Animal Center of the Academy of Military Medical Sciences, Laboratory Animal Certificate of Proficiency: 0016920.

### Establishment of NTDs mouse animal model

C57BL/6 males and females mice were mated in a ratio of 2:1 overnight, the vaginal plug was detected at 8:00 am on the following morning, which designated as E0.5 if the presence of a vaginal plug. Pregnancy test thrombi, and weighing, pregnant rats will be found in vaginal cage feeding, were randomly divided into control group and experimental group, and check the day of thrombolysis as E0.5. Pregnant mice were weighed at E7.5 of gestation, and those who gained more than 2 g were given RA 25 mg/kg gavage as the experimental group, the control group was given the same dose of edible oil gavage. On E9.5 and E10.5, pregnant mice were euthanized by cervical dislocation and embryos were dissected from decidual tissue and placed in ice-cold, DEPC-treated PBS.

### Immunohistochemical (IHC) staining

We performed IHC staining for FBXO30 and psmad-1/5 on the same paraffin-embedded tissue blocks that were used for clinical diagnosis. Immunohistochemistry was performed using the avidin–biotin complex method (Vector Laboratories), including heat-induced antigen-retrieval procedures. Incubation with antibodies against FBXO30 (1:50 dilution; Santa-Cruz), p-smad1/5 (1:100;CST) was performed at 4 °C for 18 h. Quality assessment was performed on each batch of slides by including a negative control in which the primary antibody was replaced by 10% normal goat serum to preclude nonspecific signals. The levels of FBXO30 and p-Smad1/5 were analyzed by gray-scale analysis (ZEN 2012 ZEISS COMPANY).

### Human samples

In the high-incidence areas of NTDs such as Liulin in Shanxi province,after the informed consent of enrolled pregnant and their families, NTDs samples and normal fetal nerve tissues are collected. The NTDs were classified using the International Classification of Diseases (ICD-10).The enrolled pregnant women were diagnosed by trained local clinicians using ultrasonography. The surgical procedures were performed as previously described^38^. The epidemiological method was described in detail in our previous publication^39^.

### Nano String nCounter assay

The numbers of transcripts in human embryonic brain tissue were detected by the NanoString nCounter. Total RNA exteaction followed the manufacturer’s instructions (miRNeasy Mini Kit, Qiagen) and gene-specific probes were designed by the manufacturer (NanoString Technologies). Hybridizations were carried out according to the nCounter Element XT Reagents User Manual. Totally, 100 ng of each RNA sample was mixed with 8 μL of master mix for each reaction volume of 15 μL. The hybridizations were incubated at 65 °C at least 16 h, but no more than 48 h, then eluted and immobilized in the cartridge for data collection, which was performed on the nCounter Digital Analyzer. Data were filtered using quality control (QC) criteria according to the manufacturer’s recommendations. Raw counts of QC-passed samples were normalized using three reference genes as internal controls (GAPDH, CLTC, and GUSB). QC and normalization procedures were performed using nSolver Analysis Software v2.0; data were log2-transformed before further analysis.

### Statistical analysis

All the experiments were repeated independently at least three times, and the data are presented as mean ± s.d. Statistical significance was determined using the Student’s *t* test. A *P* value of <0.05 was considered to be statistically significant and is presented as *P < 0.05 or **P < 0.01.

## Results

### Reduced FBXO30 expression correlates with human NTDs

**Fig. 1:**
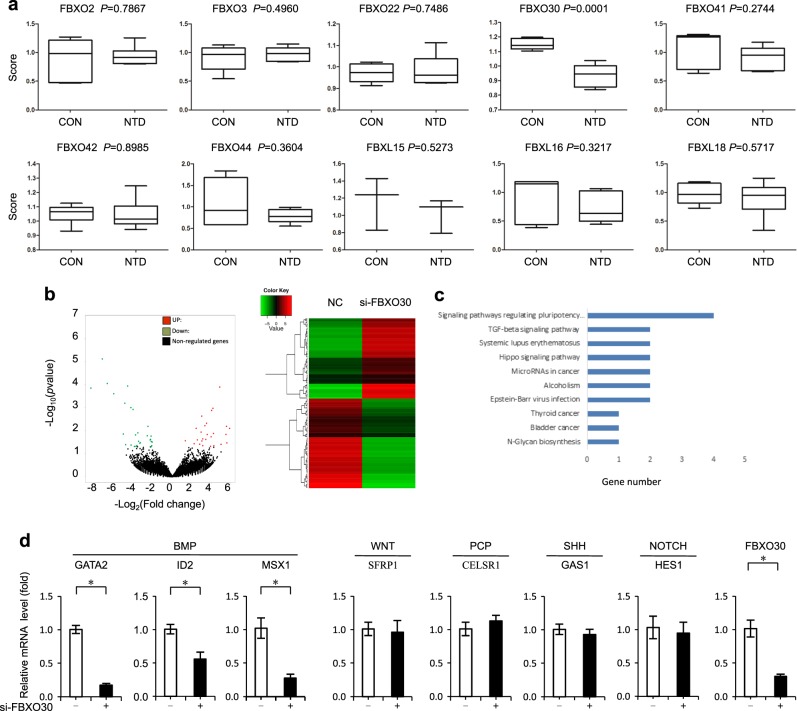
FBXO30 expression is lowered in human NTDs tissues. **a** iTRAQ ratio of F-box family for control and NTD brain tissue smaples: control samples (C2, C3, and C7); NTD samples (N5, N6, and N10). **b** SiRNA-NC vs. siRNA-FBXO30 in NT2/D1 cells. The total RNA was analyzed using RNA-seq. A total of DEGs showing differential expression were identified in the APN-treatment group (*P* < 0.05). **c** KEGG analysis of the DEGs in indicated groups. The −log10 *p* value of enrichment is shownon *x*-axis; the numbers represent the number of associated proteins for each term. **d** NT2/D1 cells were transfected with siRNA FBXO30. Expression of developmental pathway-related genes was analyzed by qPCR. All the above data are mean ± SD. (*n* = 3), *p < 0.05, by Student’s *t* test

Previously, we used a mass spectroscopy-based proteomic screen (Nano-HPLC/MS/MS) to examine differences in proteins between the brains of three fetuses with NTDs and matched normal fetuses. Strikingly, the protein level of FBXO30 was significantly downregulated in NTD tissue (Fig. 1a, S2 Tables). Other F-box proteins were not significantly changed in NTD tissue. Dysregulation of FBXO30 is, therefore, likely to pose a potential risk for NTDs. In this study, we focus on the function of FBXO30.

To test the effect of FBXO30 knockdown in NT2/D1 cells, we performed RNA-seq in control and FBXO30-depleted NT2/D1 cells. By comparing libraries from control and FBXO30-depleted cells, we identified a significant number of differentially expressed genes, including 86 upregulated and 78 downregulated genes in FBXO30-depleted cells; (Fig. 1b; S3 and S4 Tables). Further Gene Ontology (GO) and KEGG pathway analysis indicated that these differentially expressed genes were enriched for GO terms of multiple biological processes, molecular functions, and signaling pathways (Fig. S1a), and KEGG pathways of developmental biology, including signaling pathway regulating pluripotency, TGF-beta signaling pathway (Fig. 1c, Fig. S1b and S5 and S6 Tables). Previous studies have identified more than 200 candidate genes associated with NTDs, whose functions are required for neural tube closure^40^. To determine whether FBXO30 specifically regulates NTD candidate genes, the endogenous expression levels of a series of reported NTD target genes were measured by quantitative PCR analysis, including the PCP-related gene, *CELSR1*^41^, the WNT-related gene, *SFRP1*^42^, the SHH-related gene, *GAS1*^43^, the NOTCH-related gene, *HES1*^44^, and the BMP-related genes, *GATA2*, *ID2*, and *MSX1*^26,45^. Knockdown of FBXO30 reduced the mRNA levels of BMP target genes, *GATA2*, *ID2*, and *MSX1*, without significant effects on PCP-, WNT-, SHH-, and NOTCH-related genes in NT2/D1 cells (Fig. 1d). Similar results obtain from in HEK293 cells (Fig. S1c). Overexpression of FBXO30 increased the expression of *GATA2*, *ID2*, and *MSX1*, indicating that FBXO30 promotes the transcription of BMP pathway target genes (Fig. S1d). Collectively, these results indicate that FBXO30 regulates BMP pathways.

### FBXO30 ubiquitin ligase interacts with and ubiquitylates RARγ

**Fig. 2:**
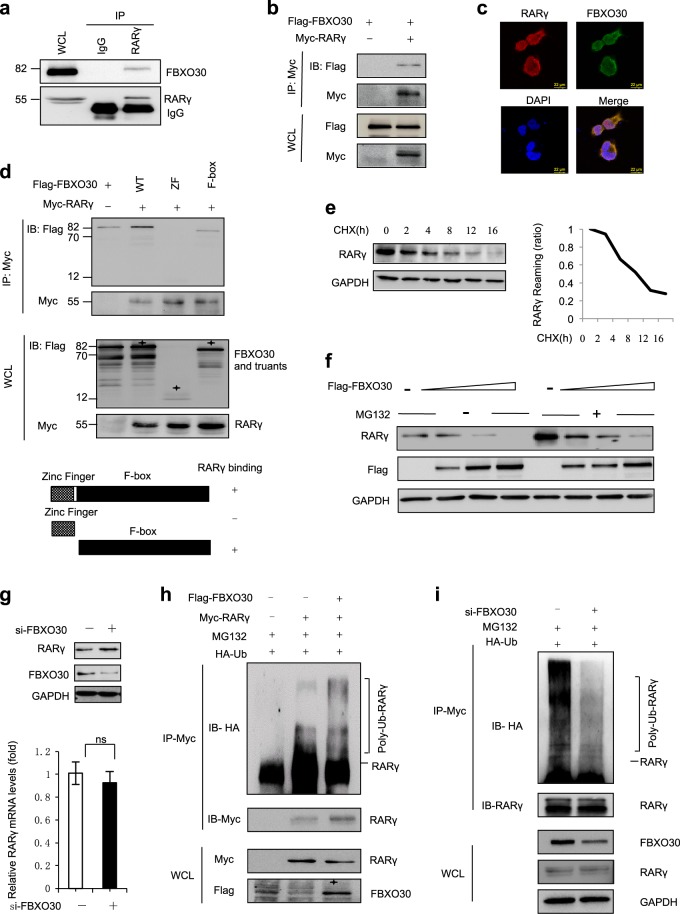
FBXO30 ubiquitin ligase interacts with and ubiquitylates RARγ. **a** The whole HEK293 cell extract were immunoprecipitated with anti-RARγ antibody and Normal rabbit IgG antibody, and analyzed by western blot with anti-FBXO30 and anti-RARγ antibodies. **b** HEK293 cells were transfected with Myc-RARγ and Flag-FBXO30 as indicated. Equal amounts of cell extract were immunoprecipitated with anti-Myc antibody, and analyzed by western blot with anti-Flag and anti-Myc antibodies. **c** The colocalization of FBXO30 and RARγ in the HEK293 cells. Direct immunofluorescence analysis was performed. Images were captured by confocal microscope and the nuclei were stained with DAPI. Scale bars, 22 μm. **d** Mapping the interacting regions of RARγ with FBXO30. HEK 293 cells were transfected with Myc-RARγ and Flag-tagged FBXO30 truncates as indicated. The cell lysates were immunoprecipitated with anti-Myc antibody and the IP samples were analyzed by western blot with Flag and Myc antibodies. The asterisks indicate the FBXO30 mutants. **e** HEK293 cells were treated with cycloheximide (50 μg/mL) for the indicated times and then harvested for western blotting. **f** HEK293 cells were transfected with increasing amounts of FBXO30. After 36 h, cells were treated with MG132 (20 μM) 12 h. Aliquots of total lysates were immunoblotted to detect anti-RARγ and anti-Flag antibody. **g** HEK293 cells were transfected with either control siRNA or siRNA targeting FBXO30. Endogenous FBXO30 and RARγ expression was analyzed by western blot (upper). Total RNA was subjected to real-time RT-PCR analysis (lower). Expression levels of FBXO30 and RARγ were determined by the comparative threshold cycle method. Mean values and SD are depicted (*n* = 3). **h** FBXO30 and RARγ were expressed in HEK293 cells together with HA-ubiquitin as indicated. The cells were treated with MG132 (20 μM) for 12 h. Myc-RARγ proteins were immunoprecipitated, followed by western blot with anti-HA antibody to indicate poly-ubiquitylated RARγ. **i** HEK293 cells were transfected with HA-Ub and siFBXO30 for 36 h and then treated with MG132 (20 μM) for 12 h. RARγ proteins were immunoprecipitated, followed by western blot with anti-HA antibody to indicate poly-ubiquitylated RARγ

IP/MS was performed using a RARγ antibody in human embryonic kidney HEK293 cells (Fig. S2a). A total of 550 unique proteins were identified with the detection of at least one unique peptide at a 1% false-discovery rate and these unique proteins were further used for subsequent KEGG enrichment analysis in DAVID. We found that the proteasome pathway might play a role in the regulation of RARγ turnover (S6 Table). Further, KEGG pathway analysis indicated that these proteins that interact with RARγ were enriched in the proteasome pathway and one of the interacting proteins was the F-box protein, FBXO30 (Fig. S2b and S2C, S7 Table). We next performed IP-western assays to validate whether the interaction between RARγ and FBXO30 occurs in a physiological context. Endogenous RARγ was easily co-immunoprecipitated with endogenous FBXO30, but not by a control IgG in HEK293 cells (Fig. 2a). Indeed, RARγ was co-immunoprecipitated with ectopic FBXO30 (Fig. 2b). We also detected interaction between other RARs members RARα or RARβ and FBXO30 occurs in a physiological context. However, endogenous RARα and RARβ fail to interact of FBXO30 (Fig. S2d). RARγ, as a nuclear receptor, is located both in the nucleus and the cytoplasm. FBXO30 is mainly located in the cytoplasm as a ubiquitin protein ligase. Indirect immunofluorescence assays revealed that RARγ and FBXO30 were colocalized predominantly in the cytoplasm of HEK293 cells (Fig. 2c). Similar results were also obtained in NT2/D1 embryonal carcinoma cells (Fig. S2e). Human FBXO30 contains an F-box domain (aa 373–409) and a zinc finger domain (aa 48–109). To further clarify the interaction region between FBXO30 and RARγ, we constructed truncated FBXO30 proteins, FBXO30-ZF (1–109) and FBXO30-F-box (110–745). Truncation interaction experiments revealed that the F-box domain of FBXO30 was sufficient and necessary for the interaction (Fig. 2d). Together, these data demonstrate that FBXO30 interacts with RARγ endogenously in cultured cells, and that the F-box domain of FBXO30 mediates their interaction.

Ubiquitylation usually mediates the protein turnover by the proteasome; however, it is not known whether RARγ can be ubiquitylated. Half-life analysis showed RARγ to be a relatively short-lived protein with a half-life of 4 h (Fig. 2e). It has been reported that F-box proteins contain additional protein interaction domains that bind ubiquitylation targets^46,47^. Next, we investigated whether FBXO30 functions as an E3 ligase of RARγ for ubiquitylation and degradation. The level of RARγ was significantly decreased in the presence of FBXO30 in a dose-dependent manner, and this effect was blocked by the proteasome inhibitor MG132 (Fig. 2f). Knockdown of endogenous FBXO30 elevated the steady-state level of endogenous RARγ, but had no significant effect on the mRNA abundance of RARγ (Fig. 2g, Fig. S2f). Overexpression of FBXO30 dramatically promoted the polyubiquitylation of RARγ (Fig. 2h). Knockdown of FBXO30 significantly reduced the poly-ubiquitination of RARγ (Fig. 2i). Overexpression of FBXO30 or knockdown of endogenous FBXO30 had no significant effect on level of endogenous RARα and RARβ (Fig. S2g and S2h). It suggested that FBXO30 selective interact with RARγ and regulate expression of RARγ. These results indicate that FBXO30 can promote ubiquitin-mediated proteasomal degradation of RARγ.

### FBXO30 promotes BMP pathway activity

**Fig. 3:**
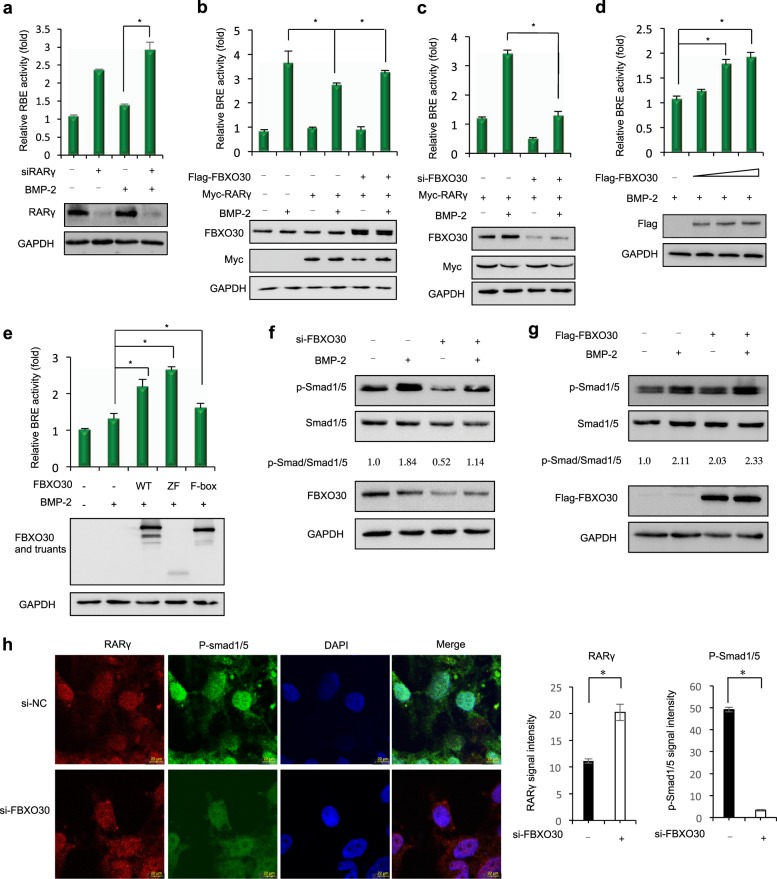
FBXO30 promotes BMP pathway activity. **a** HEK293 cells were transfected with siRNA against RARγ as indicated. Thirty-six hours after transfection, cells were treated with BMP-2 (100 ng/ml) for 12 h before BRE-luciferase activity was measured. Data are mean ± s.d. (*n* = 3). **b** HEK293 cells were transfected with the FBXO30 together RARγ as indicated. Thirty-six hours after transfection, cells were treated with BMP-2 (100 ng/ml) for 12 h before BRE-luciferase activity was measured. Data are mean ± s.d. (*n* = 3). **c** HEK293 cells were transfected with the siRNA-FBXO30 and RARγ as indicated. Thirty-six hours after transfection, cells were treated with BMP-2 (100 ng/ml) for 12 h before BRE-luciferase activity was measured. Data are mean ± s.d. (*n* = 3). **d** HEK293 cells were transfected with the increasing amounts of FBXO30. Thirty-six hours after transfection, cells were treated with BMP-2 (100 ng/ml) for 12 h before BRE-luciferase activity was measured. Data are mean ± s.d. (*n* = 3). **e** HEK293 cells were transfected with the FBXO30 WT or deletion mutants as indicated. Thirty-six hours after transfection, cells were treated with BMP-2 (100 ng/ml) for 12 h before BRE-luciferase activity was measured. Data are mean ± s.d. (*n* = 3). **f**, **g** Overexpression of FBXO30 or siRNA-FBXO30 transfected in HEK293 cells as indicated. Thirty-six hours after transfection, cells were treated with BMP-2 (100 ng/ml) for 12 h before cell lysates harvest. Western blot was performed to examine the pSmad1/5 and total Smad1/5 levels in whole-cell lysates. Aliquots of total lysates were immunoblotted to indicate antibody. Numbers at the bottom were generated by quantification (Image J) of the pSmad1/5 signal normalized to the Smad1/5 signal. **h** siRNA-FBXO30 or siRNA-control was transfected into HEK293 cells. Direct visualization or indirect immunofluorescence was performed. Scale bars, 22 μm

Overexpression of RARγ in chicken embryonic neural tube inhibits the BMP pathway, indicating that RARγ negatively regulates the BMP pathway and participates in the process of neural tube closure^27^. We, therefore, sought to better characterize the functional relevance of FBXO30 and RARγ for BMP signaling. BMP-2 treatment remarkably stimulated BMP-responsive BRE-luc activity and knockdown of RARγ enhanced it (Fig. 3a). Overexpression of RARγ inhibited BRE-luc activity. When RARγ was coexpressed with FBXO30, FBXO30 substantially antagonized the inhibitory effect of RARγ (Fig. 3b, column 6 versus column 4). Knockdown of endogenous FBXO30 significantly reduced stimulation of BRE activity (Fig. 3c, column 4 versus column 2). Furthermore, FBXO30 significantly inhibited the activity of endogenous BRE-luc activity in a dose-dependent manner (Fig. 3d). Interestingly, both F-box domain and the zinc finger domain had a significant effect on BRE-luc activity (Fig. 3e), indicating that FBXO30 regulated BRE-luc activity through zinc finger-associated mechanisms.

BMP-Smad1/5 is a key regulatory molecule in the development of the nervous system. RA/RAR represses BMP signal duration by reducing the level of phosphorylated Smad1 (pSmad1)^27^. Through this mechanism, RAR activation contributes to shut off BMP signaling. Knockdown of RARγ significantly enhanced the protein levels of phosphorylated Smad1/5 (Fig. S3a). Depletion of FBXO30 attenuated Smad1/5 phosphorylation (Fig. 3f). Overexpression of FBXO30 increased p-Smad1/5 levels (Fig. 3g). Immunofluorescence showed that knockdown of FBXO30 increased RARγ levels and decreased p-Smad1/5 levels (Fig. 3h and Fig. S3b). Collectively, these data suggest that FBXO30 is a positive regulator of BMP signaling in cultured mammalian cells, at least partially through directing the degradation of RARγ.

### RA promotes the ubiquitylation reduces the levels of FBXO30

**Fig. 4:**
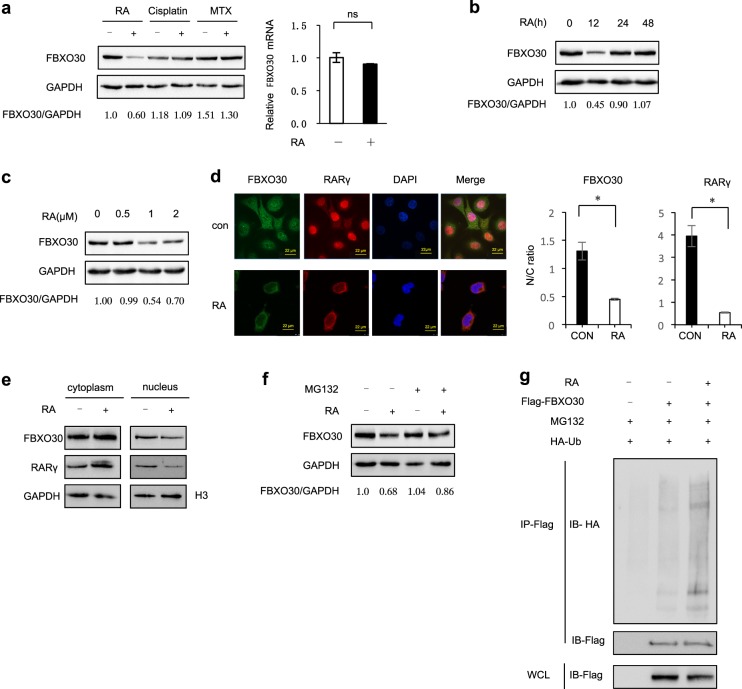
RA treatment promotes to ubiquitylate FBXO30. **a** HEK293 cells were treated with RA (1 μM, 12 h) or Cispaltin (100 μmol/L) or MTX (1 μM) for the indicated times. Cell lysates were harvested to detect FBXO30 level (left). Real-time PCR analysis showed no effect of RA on FBXO30 mRNA level (right). Mean values and SD are depicted. **b** HEK293 cells were treated with RA (1 μM) for the indicated time. Cell lysates were harvested to detect FBXO30 level. **c** HEK293 cells were treated with RA 12 h for the indicated concentration. Cell lysates were harvested to detect FBXO30 level. **d** HEK293 cells were treated with RA (1 μM) for 12 h. Direct visualization or indirect immunofluorescence was performed. Scale bars, 22 μm. **e** HEK293 cells were treated with RA (1 μM) for 12 h. Distribution of endogenous FBXO30 in HEK293 cells was determined by cell fractionation. The fractions were subjected to western blot with the indicated antibodies. H3 is a nucleolar marker protein. **f** HEK293 cells were treated with RA (1 μM) and/or MG132 for 12 h. Aliquots of total lysates were immunoblotted to detect anti-FBXO30 antibody. **g** HEK293 cells transfected with HA-Ub and FBXO30 were treated with MG132. After RA treatment, the ubiquitylation of FBXO30 was analyzed

Vitamin A, and its biologically active metabolite, RA, are thought to be involved in neural tube patterning^2^. We found abnormal expression of FBXO30 in NTD tissues; therefore, we investigated the potential effect of FBXO30 on environmental factors. First, we examined the protein level of FBXO30 under RA treatment in mammalian cells. The protein level of FBXO30 was significantly decreased both in HEK293 and NT2/D1 cell lines under RA treatment (Fig. 4a, left, lanes 1–2 and Fig. S4a, left, lanes 1–2). This effect was specific because FBXO30 levels were not affected by treatment with cisplatin, which induces DNA damage, or MTX (methotrexate, a folate antagonist). RA had no significant effects on FBXO30 mRNA levels (Fig. 4a, right), indicating that FBXO30 protein might be stable under stress. Next, we found that 1 μM RA treatment reduced the expression of FBXO30 after 12 h in HEK293 cells (Fig. 4b, c). Similar results obtained from NT2/D1 cell (Fig. S4b and c). As illustrated in Fig. 2c, FBXO30 both existed in the nucleus and cytoplasm, while RA treatment caused FBXO30 to shuttle from the nucleus to the cytoplasm. The cytoplasmic translocation of FBXO30 was also quantified by calculating N/C (nuclear/cytoplasm) intensity ratios from stained cells (Fig. 4d). We also analyzed distribution of FBXO30 protein in HEK293 cells. These results also showed that FBXO30 protein shuttled from the nucleus to the cytoplasm upon RA treatment (Fig. 4e). To determine whether RA-induced FBXO30 degradation is mediated by the proteasomal pathway, we treated HEK293 cells with the proteasome inhibitor, MG132, and found that the RA-induced decrease in FBXO30 protein can be readily restored by MG132 (Fig. 4f). Next, we sought to determine whether RA-mediated FBXO30 degradation is a consequence of ubiquitination. The results showed that RA promoted the polyubiquitylation of FBXO30 (Fig. 4g). Collectively, these results demonstrate that RA promotes FBXO30 degradation via ubiquitylation-mediated proteasomal degradation.

### FBXO30 antagonizes the effect of RA on BMP signaling

**Fig. 5:**
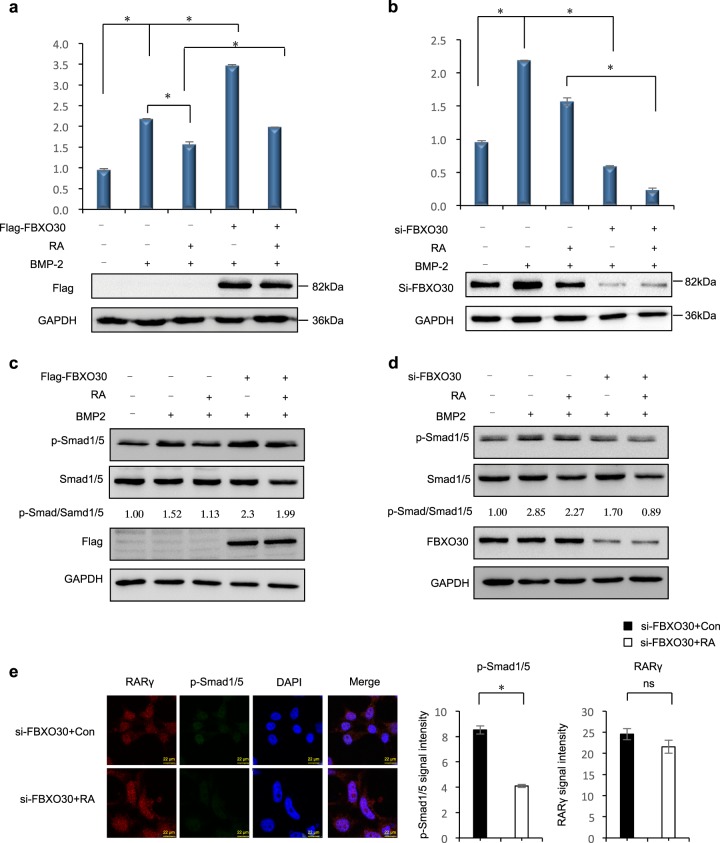
FBXO30 antagonizes the effect of RA on BMP signaling. **a** HEK293 cells were transfected overexpression of FBXO30. Thirty-six hours after transfection, BRE-luciferase activity was measured after treatments with BMP-2 (100 ng/ml) and/or RA (1 μM) for 12 h. Data are mean ± s.d. (*n* = 3). **b** HEK293 cells were transfected with the siRNA-FBXO30. Thirty-six hours after transfection, BRE-luciferase activity was measured after treatments with BMP-2 (100 ng/ml) and/or RA (1 μM) for 12 h. Data are mean ± s.d. (n = 3). **c**, **d** Overexpression of FBXO30 or siRNA-FBXO30 transfected in HEK293 cells as indicated. Thirty-six hours after transfection, cells were stimulated with BMP-2 and/or RA for 12 h before harvesting. Western blot was performed to examine the pSmad1/5 and total Smad1/5 levels in whole-cell lysates. Aliquots of total lysates were immunoblotted to indicate antibody. Numbers at the bottom were generated by quantification (Image J) of the pSmad1/5 signal normalized to the Smad1/5 signal. **e** siRNA-FBXO30 or siRNA-normal transfected in HEK293 cells as indicated. Thirty-six hours after transfection, cells were stimulated with BMP-2 and/or RA for 12 h before harvesting. Direct visualization or indirect immunofluorescence was performed. Scale bars, 22 μm

RA represses BMP signaling by reducing the level of phosphorylated Smad1 (pSmad1). During neural development in the chicken embryo, the RA/RAR pathway also suppresses BMP-regulated proliferation and differentiation of neural progenitor cells^27^ (Fig. S5a). We, therefore, asked if FBXO30 expression affected the RA repression of BMP signaling. RA treatment strongly inhibited BMP2-induced, as well as basal, BRE-luc activities (Fig. 5a lane 2 vs. lane 3), whereas the inhibitory activity of RA was markedly enhanced in cells overexpressing FBXO30. Furthermore, depletion of FBXO30 significantly decreased BRE-luc activities after RA treatment (Fig. 5b).

We then examined pSmad1/5 levels in response to BMP-2 and/or RA in cells overexpressing or depleted for FBXO30. We found that RA-reduced BMP2-activated Smad1/5 phosphorylation, but did not affect the overall amount of Smad1/5 in whole-cell lysates, consistent with previous studies (Fig. 5c, lane 2 vs. lane 3). Furthermore, overexpression of FBXO30 restored pSmad1/5 levels upon RA treatment (Fig. 5c, lane 3 vs. lane 5). Depletion of FBXO30 enhanced the decrease in pSmad1/5 levels after RA treatment (Fig. 5d, lane 3 vs. lane 5). Consistently, immunofluorescence experiments also showed that RA decreased the p-Smad1/5 level after knockdown of FBXO30 (Fig. 5e and Fig. S5b). Collectively, we conclude that FBXO30 antagonizes the RA-mediated inhibition of BMP signaling by promoting BRE-luc activities and pSmad1/5.

### RA reduced the levels of FBXO30 in NTD mice

**Fig. 6:**
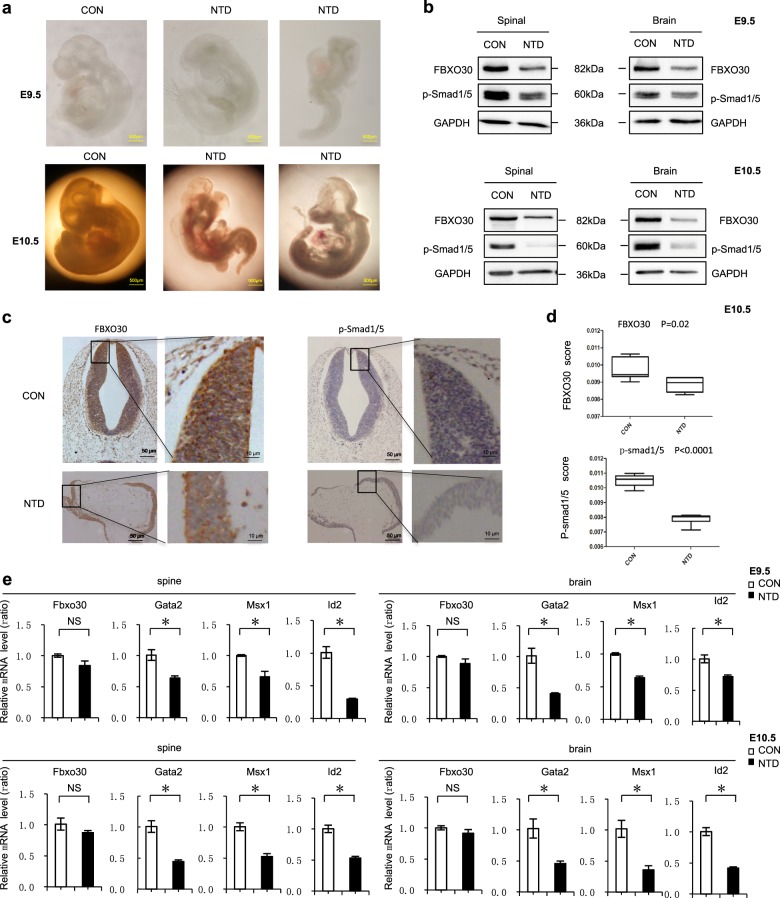
RA caused reduced levels of FBXO30 at BMP target genes. **a** RA-induced schizencephaly in C57BL/6 mouse embryos at 9.5 and 10.5 day. **b** Cranial neural tissue of normal and RA-induced mouse NTDs was harvested at E9.5 and E10.5, and analyzed by western blotting. Aliquots of total lysates were immunoblotted to indicate antibody. **c** Representative images from immunohistochemical staining of FBXO30 and pSmad1/5 in the cranial neural tissue from E10.5. **d** FBXO30 and pSmad1/5 expression scores are shown as box plots, with the horizontal lines representing the median. Data are mean ± s.d. (*n* = 3), *p < 0.05, by Student’s *t* test. **e** Fbxo30 and BMP target genes Gata2, Id2, and Msx1 mRNA in cranial neural tissue of RA-induced mouse NTDs from E9.5 and E10.5 was measured by RT-qPCR. Data are mean ± s.d. (*n* = 3)

RA is a well-known teratogen when administered to embryos and induces NTDs, including spina bifida, exencephaly and anencephaly in several different species^48–50^. We employed a modified rapid RA-induced NTD mouse model via gavage of excess RA (25 mg/kg) at E7.5^51^. As shown in Fig. 6a, normal mice appeared full and rounded, and the neural tube was completely closed (E10.5). Malformed embryos had different degrees of developmental delay; embryos were small, and the main phenotype was NTDs. The FBXO30 level was remarkably decreased in RA-induced cranial neural tissue from E9.5 and E10.5 embryos, while the level of p-Smad1/5 was also decreased (Fig. 6b). We next examined FBXO30 and p-Smad1/5 levels in three cranial neural tissue samples and matched normal tissues by IHC analysis. The staining of total FBXO30 and p-Smad1/5 was decreased in mouse NTD samples compared with normal tissues (Fig. 6c, d). Similar results obtained between affected and nonaffected areas of the neural tube in the NTD model embryos. The staining of total FBXO30 and p-Smad1/5 was also decreased in affected areas of mouse NTD samples compared with nonaffected areas. (Fig. S6a, b). We then evaluated changes in expression of BMP-target genes at E9.5 and E10.5. qPCR assays were performed on cranial neural tissue of E9.5 and E10.5 mouse embryos. The mRNA levels of BMP pathway target genes, *Gata2*, *Id2*, and *Msx1*, were significantly reduced in NTD embryos compared to those in controls (Fig. 6e). The mRNA level of FBXO30 expression was no significant difference both in brain and spinal tissues from NTD embryos, consistent with the previously described cytological effects in HEK293 cells. Together, these results indicate that the downregulation of FBXO30 expression is related to the occurrence of RA-induced mouse NTDs, probably through regulating alterative BMP signaling activity.

### Levels of FBXO30 and BMP target gene expression are decreased in high-retinoid content NTD fetuses

**Fig. 7:**
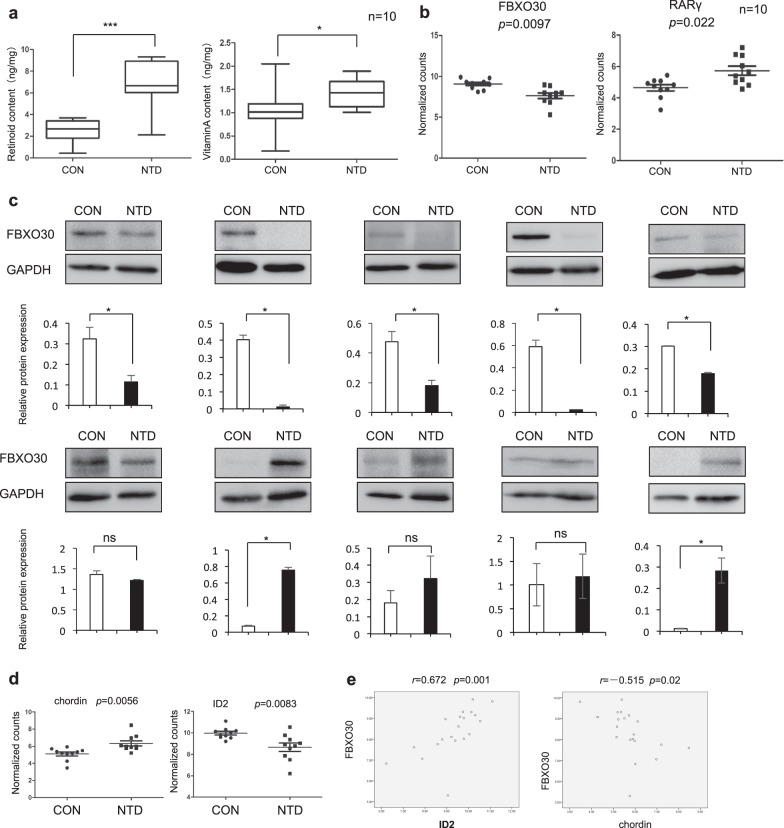
FBXO30 downregulated with decreased BMP related gene expression in high-retinoid NTDs. **a** Retinoid and Vitamin A contents in brain tissue of normal fetus and fetus with NTDs. **b** The mRNA expression of FBXO30 and RARγ in the brain of NTD fetuses, determined by Nano String. Data are mean ± s.d. (*n* = 10), *p < 0.05, by Student’s *t* test. **c** Detection of FBXO30 in brain tissues from human anencephaly and normal cases by western blot. Total GAPDH was used as a loading control. Data are mean ± s.d. (*n* = 3), *p < 0.05, by Student’s *t* test. **d** The mRNA expression of BMP related Genes in the brain of NTD fetuses, determined by Nano String. Data are mean ± s.d. (*n* = 10), *p < 0.05, by Student’s *t* test. **e** Pearson’s correlation analysis between FBXO30 expression and ID2, chordin expression

Numerous environmental factors and gene–environment interactions have been strongly implicated in NTD etiology^4^. To explore the potential involvement of RA on abnormal expression of FBXO30 and BMP signaling in NTDs, we tested high-retinoid NTD fetuses. Ten pairs of normal and anencephaly fetal brains were selected, based on best match for age and gender, as summarized in Table 1. Levels of retinoid and vitamin A in tissues from anencephaly fetuses were higher than those in controls (Fig. 7a and Table 1). Among all fetal brain tissue pairs, the technique of nanostring results showed that FBXO30 was downregulated and RARγ upregulated in anencephaly brain samples (*p* = 0.0097, Fig. 7b). Western blot analysis of ten anencephaly subjects compared with age and gender-matched controls revealed that FBXO30 levels were decreased in most anencephaly samples ranging from 10 to 80% (Fig. 7c). These results demonstrate the downregulation of FBXO30 levels with elevated retinoid content in NTD fetuses.

**Table 1:** Clinical manifestations of normal fetus and NTD fetus

Sample ID	Clinical phenotype	Tissue	Gender	Gestational weeks	Retinoid content (ng/mg)	Vitamin A content (ng/mg)
1-1	Normal	Brain	Female	25	0.98	0.98
2-1	Normal	Brain	Male	18	3.36	0.90
3-1	Normal	Brain	Female	24	0.43	1.09
4-1	Normal	Brain	Female	18	2.33	0.84
5-1	Normal	Brain	Female	17	3.70	0.17
6-1	Normal	Brain	Male	20	3.60	0.89
7-1	Normal	Brain	Female	20	2.67	2.05
8-1	Normal	Brain	Male	20	2.11	1.18
9-1	Normal	Brain	Female	22	2.75	1.22
10-1	Normal	Brain	Female	18	2.68	1.05
1-2	Brain	Female	25	6.97	1.67	
2-2	Anencephaly	Brain	Male	18	8.79	1.68
3-2	Anencephaly	Brain	Female	24	6.04	1.15
4-2	Anencephaly	Brain	Female	18	9.31	1.53
5-2	Anencephaly	Brain	Female	16	6.13	1.33
6-2	Anencephaly	Brain	Male	20	9.27	1.12
7-2	Anencephaly	Brain	Female	20	2.12	1.00
8-2	Anencephaly	Brain	Male	20	6.35	1.89
9-2	Anencephaly	Brain	Female	21	6.00	1.13
10-2	Anencephaly	Brain	Female	20	7.68	1.54

Next, we detected BMP pathway-related genes in NTD samples, including BMP pathway-related receptors, *ActRII*, *ALK2*, *ALK3*, and *ALK6*; BMP ligands, *BMP2* and *BMP4*; BMP pathway inhibitors, *chordin* and *LRP2*; BMP pathway target genes, *GATA2*, *ID1*, *ID2*, and *noggin*; BMP pathway downstream effector molecules, *Smad1*, *Smad4*, *Smad5*, *Smad8*; and other BMP pathway-related genes, such as *Smurf1* and *Smurf2*. The results showed that expression of *chordin* was significantly increased and *ID2* was significantly decreased (Fig. 7d) (*p* < 0.05). There was no statistically significant difference in the changes of other BMP pathway-related genes (S7 Fig). Correlation analysis showed that there was a significant positive correlation between FBXO30 and the BMP pathway target gene, ID2 (*r* = 0.672, *p* < 0.05), while FBXO30 was significantly negatively related to the BMP pathway inhibitory factor, chordin (*r* = −0.515, *p* < 0.05) (Fig. 7e). These results indicate that FBXO30 levels are altered with decreased BMP target gene expression in high-retinoid content NTD fetuses.

## Discussion

Abnormal expression of RARs at critical times in nervous system development can cause serious neurodevelopmental defects. In this study, we show that FBXO30 can interact with RARγ and promote its ubiquitin-mediated degradation. Furthermore, FBXO30 participates in the regulation of BMP signaling. We also found that in mouse NTD models and human NTD specimens with RA overdose decreased FBXO30 expression was accompanied by aberrant BMP gene expression.

RARs are important nuclear transcription factors whose protein levels are critical during embryonic development. Knockout of RARs results in severe developmental defects, including absence of posterior rhabdomodes, an anomalous extension of the anterior ridge, and vertebrate malformations^52–55^. However, it has yet to be determined how RARγ is degraded by the proteasome and which E3 ligase targets RARγ for degradation. Our study was based on IP/MS data that identified a new RARγ-ubiquitin protein ligase, FBXO30. FBXO30, which is known as an E3 ubiquitin–protein ligase, mediates the ubiquitination of multiple proteins, including spindle kinesin (EG5)^56^ and some muscle-associated protein, such as skeletal muscle^57^, leading to their degradation by the proteasome. In this report, we found that FBXO30 stimulates RARγ polyubiquitylation and degradation by the proteasome under the physiological conditions of the cells, indicating that FBXO30 functions as a ubiquitin E3 ligase for RARγ. Thus this is the first report showing that an E3 ligase targets RARγ for degradation. During the ubiquitination-proteasome-mediated degradation of RARγ, the F-box domain (48–109 amino) of FBXO30 is critical for RARγ binding.

RARγ can negatively regulate the BMP pathway. On this basis, we found that FBXO30 attenuates the effect of RARγ on BMP signaling to positively regulate the BMP pathway. In vertebrates, BMPs are key signaling molecules that are employed throughout neural development to control the intricate processes involved in generating a functional CNS. Signaling of Smads 1/5/8 in response to the BMP family of proteins is essential in the development of the CNS^58^, and we also found that FBXO30 promoted the level of pSmad1/5. Recently, FBXL15 and FBXO3 were shown to stimulate the BMP pathway^36,45^. However this mainly influences neural tube development by degrading the protein level of Smurf1. In addition, FBXO45 and FBXO38 of the FBXO subfamily play a regulatory role in embryonic neural differentiation and migration^59,60^. Our studies identify a mechanism for BMP signaling that is dependent upon FBXO30. Whether other F-box family proteins are involved in embryonic neural development remains to be investigated. Elucidation of their highly specific tissue distribution and physiological functions may contribute to knowledge of the spatial and temporal complexity of nervous system development diseases.

RA is a key-signaling molecule that regulates and mediates many fundamental biological processes, through its nuclear receptor^9,17,61,62^. In the nervous system, dynamically controlled RA metabolism is critical for neuronal differentiation, brain development, and patterning. We found that in response to RA treatment, the protein level of FBXO30 was significantly downregulated, but the level of FBXO30 mRNA was not affected indicating that FBXO30 protein might be stabilized by RA treatment. Interestingly, we observed that RA triggered the accumulation of FBXO30 in the cytoplasm. Furthermore, we found that RA treatment stimulated FBXO30 polyubiquitylation together with the reduction of FBXO30 protein level. Overexpression of FBXO30 enhanced BMP signaling, which is relatively insensitive to RA. This raised the possibility that FBXO30 expression is responsible for resistance of BMP signaling to RA inhibition. We mainly focused on FBXO30 effects on physiology. We found that expression of FBXO30 and BMP target genes was almost eliminated in RA-induced mouse NTD embryos. ITRAQ MS analysis showed that abnormal expression of F-box proteins was detected in human NTD tissues. Furthermore, we observed that FBXO30 expression was significantly downregulated in NTD tissues with high-RA levels compared with normal tissues. An analysis of ten pairs of human NTD fetuses demonstrated that BMP target genes were significantly downregulated in NTD tissues with high-RA levels. Importantly, we observed a positive correlation of FBXO30 levels with BMP target genes, *ID2*, and *chordin*^26^. It is suggested that decline of FBXO30 levels accompanied by inhibition of the BMP pathway may be involved in the occurrence of high RA levels in human NTDs. Vitamin A is unique among the vitamins in that its concentration must be within a very narrow range to avoid both deficiency and toxicity. Thus, adding vitamin A or RA to embryos can easily induce teratogenic effects including major alterations in organogenesis^13^.

Taken together, this study has revealed that FBXO30 negatively regulates the protein level of RARγ. Furthermore, FBXO30 antagonizes the inhibitory effect of RA/RARγ on BMP signaling, indicating a molecular framework for FBXO30 function in unstressed and RA treated cells. It also provides the first evidence of abnormal expression of FBXO30 and BMP signaling in human NTDs with elevated levels of RA. These findings contribute to our understanding of the complexity of NTDs.

## Supplementary information

Supp Figures

Supp informations

Supp Table 1

Supp Table 2

Supp Table 3

Supp Table 4

Supp Table 5

Supp Table 6

Supp Table 7

Supp Table 8
