# Supplementary material for: F-box protein FBXO30 mediates retinoic acid receptor γ ubiquitination and regulates BMP signaling in neural tube defects
Source: Cell Death Dis. 2019 Jul 18;10(8):551. doi: 10.1038/s41419-019-1783-y (PMC6639381; doi:10.1038/s41419-019-1783-y)

Supp Figure 1

A

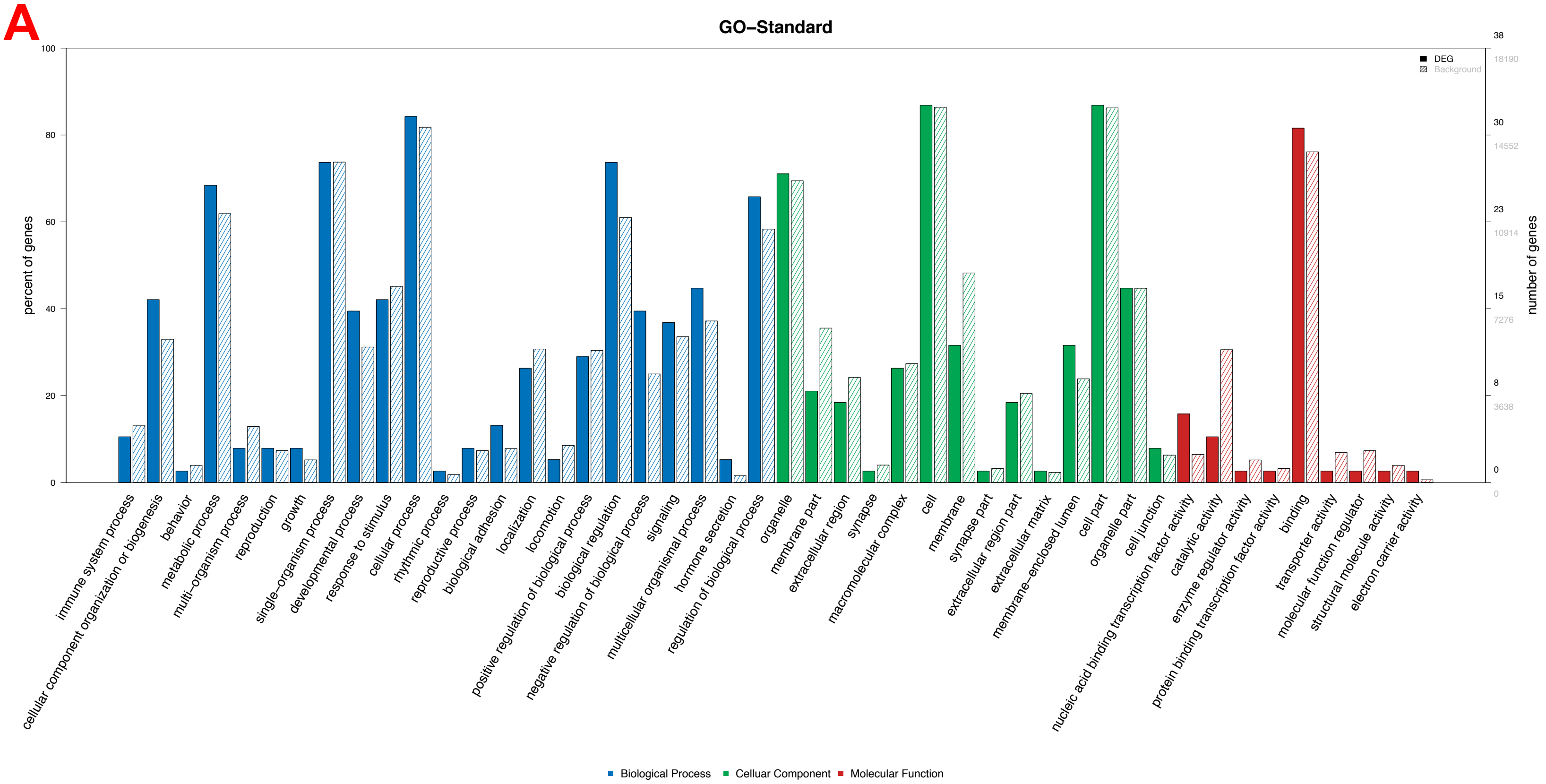

B

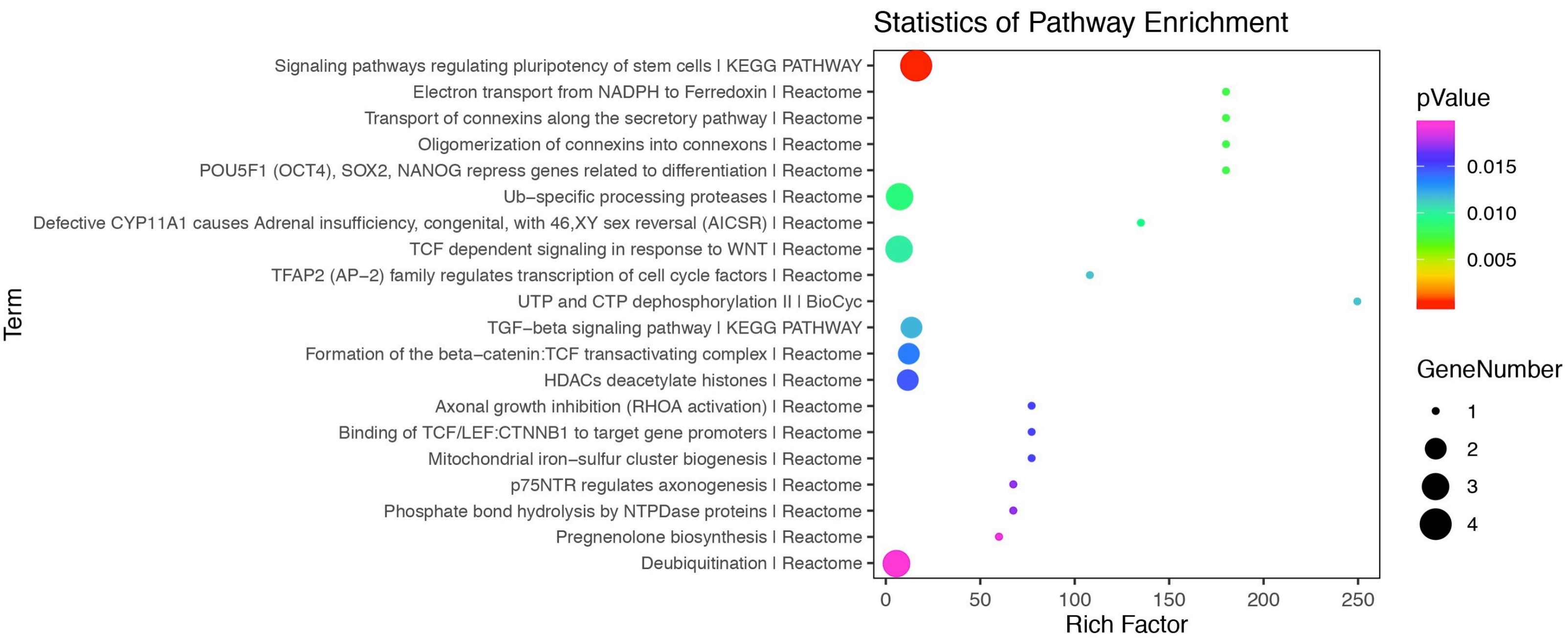

C

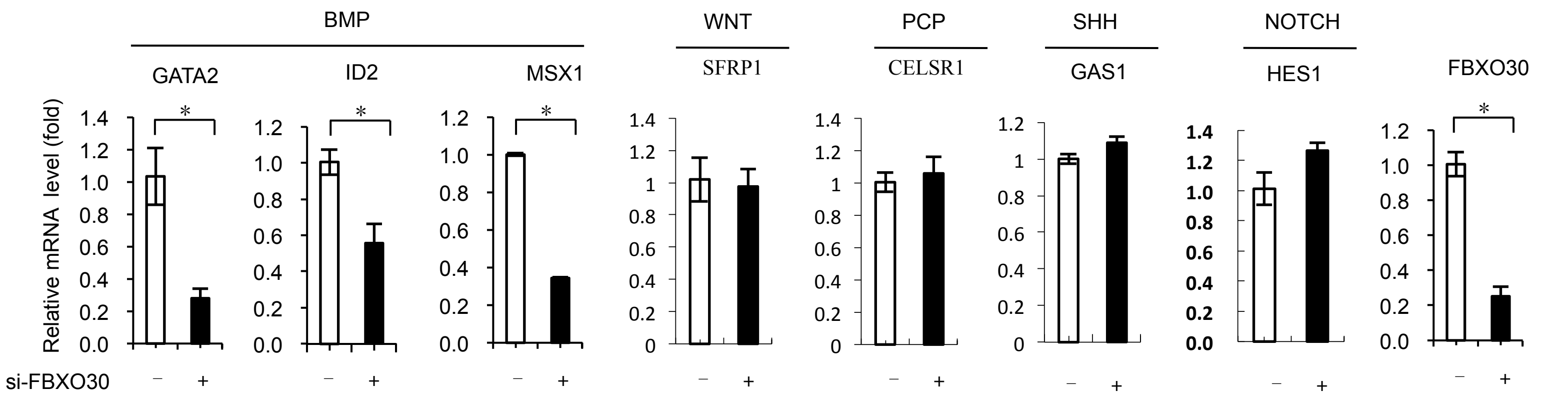
BMP
WNT
PCP
SHH
NOTCH
GATA2
ID2
MSX1
SFRP1
CELSR1
GAS1
HES1
FBXO30
Relative mRNA level (fold)
si-FBXO30
−
+
*

D

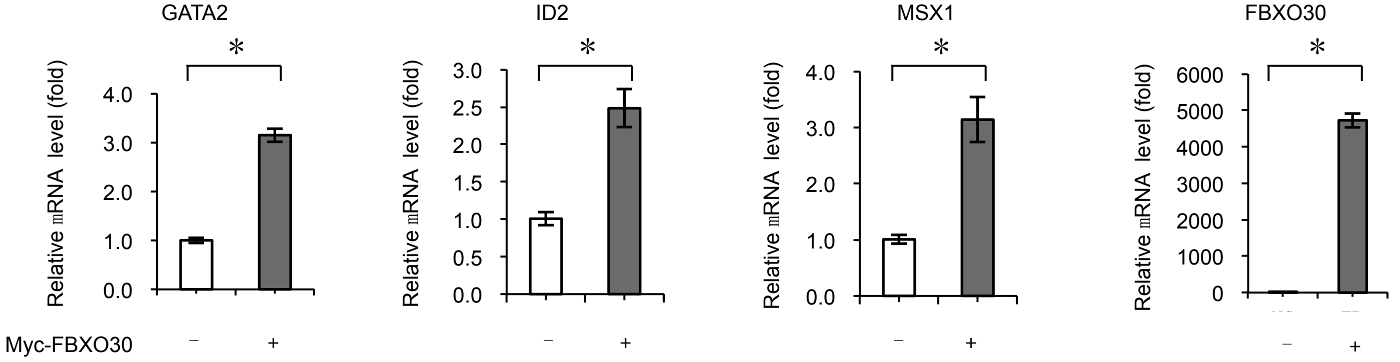
GATA2
ID2
MSX1
FBXO30
Relative mRNA level (fold)
Myc-FBXO30
−
+
*

Supp Figure 2

A

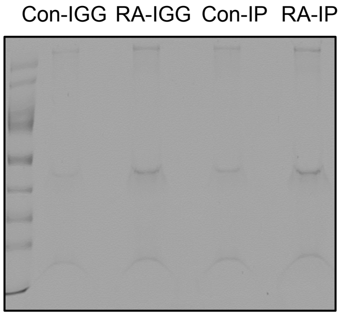

B

UniProtKB AC/ID	gene symbol	LFQ intensity IP
Q8BJL1	FBXO30	1133200

C

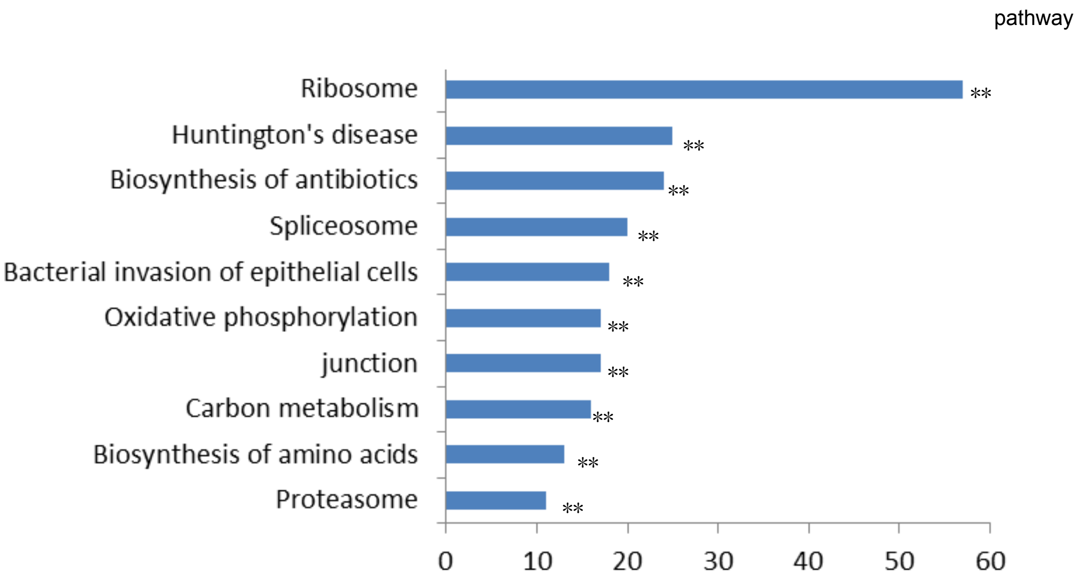

D

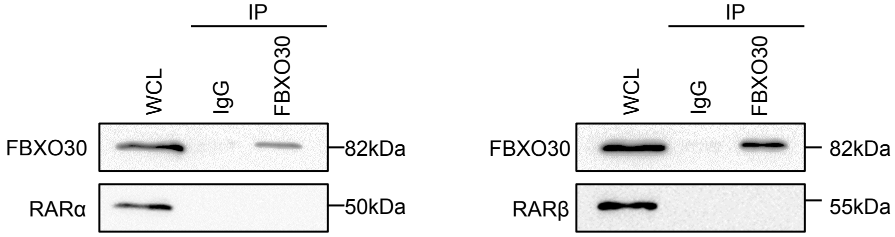

E

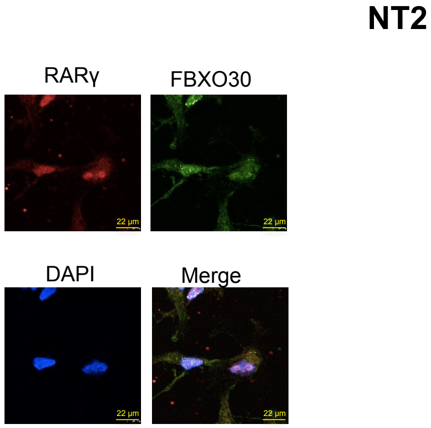

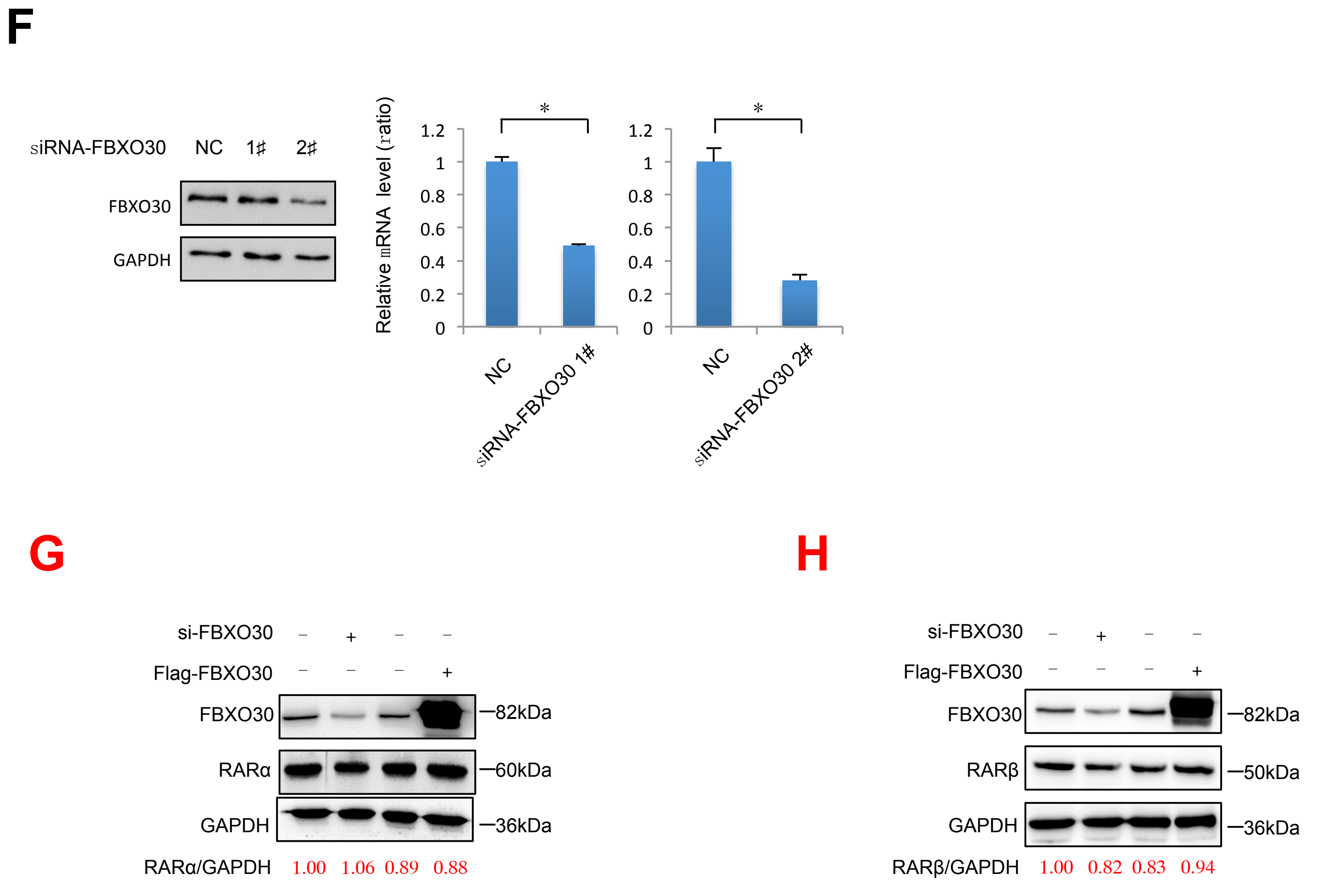

Supp Figure 3

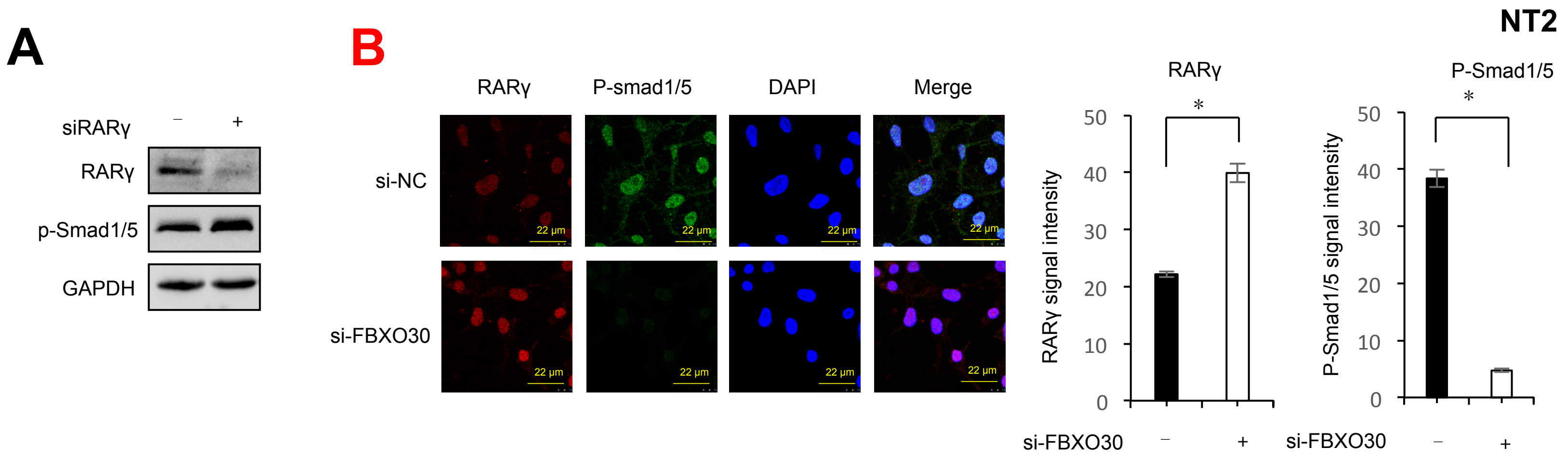

Supp Figure 4

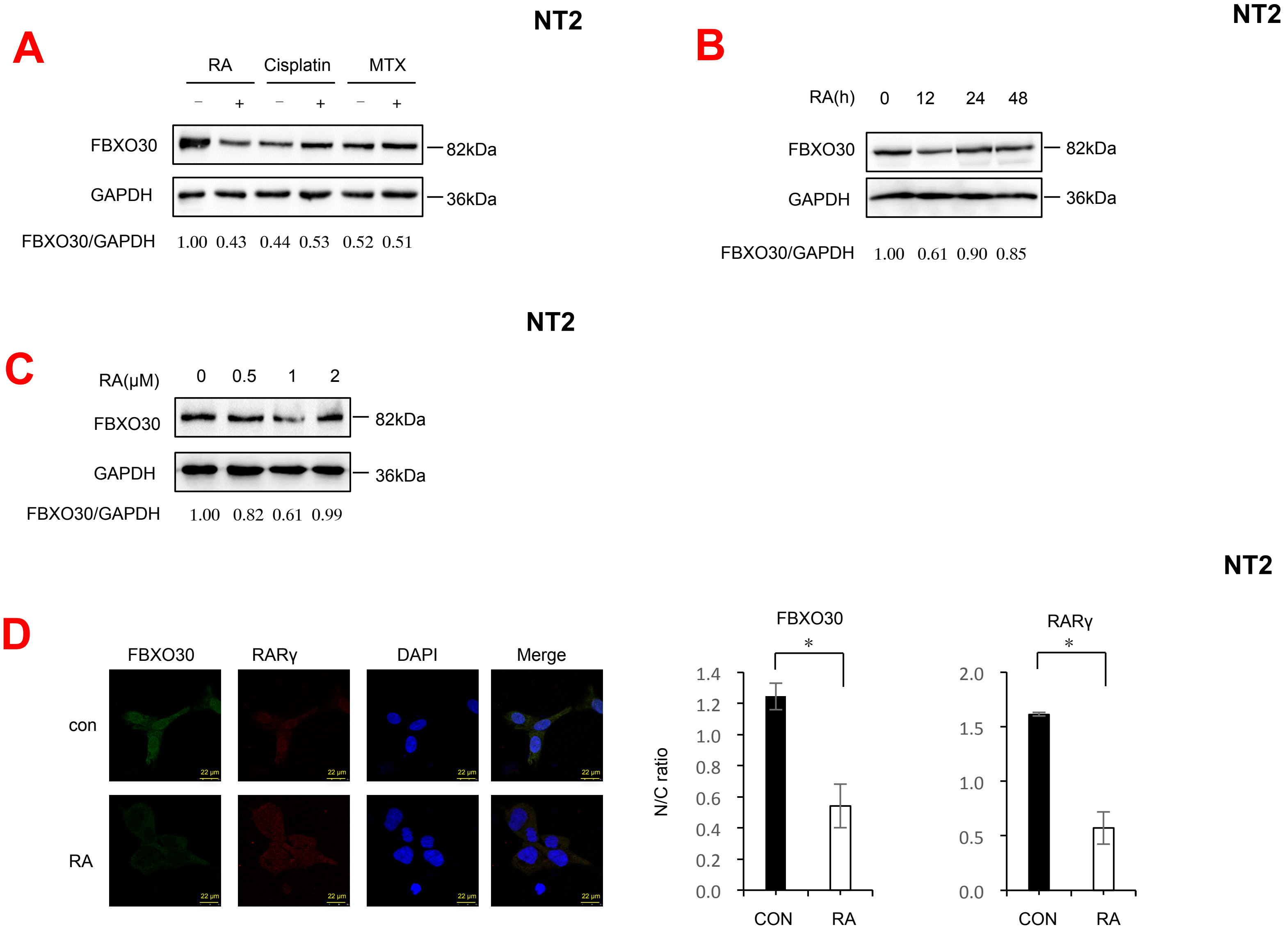

Supp Figure 5

A

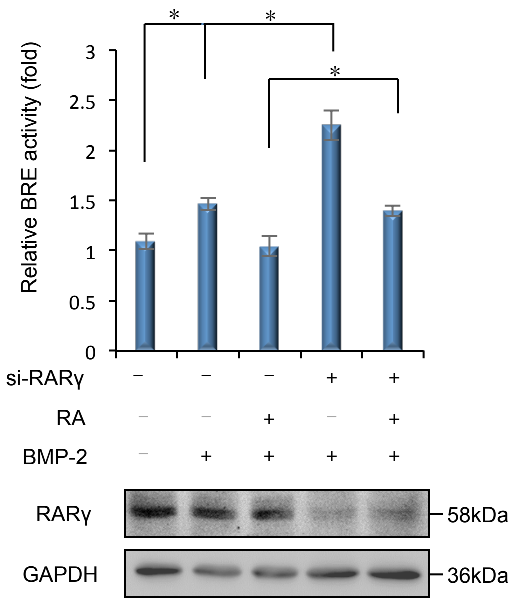

B

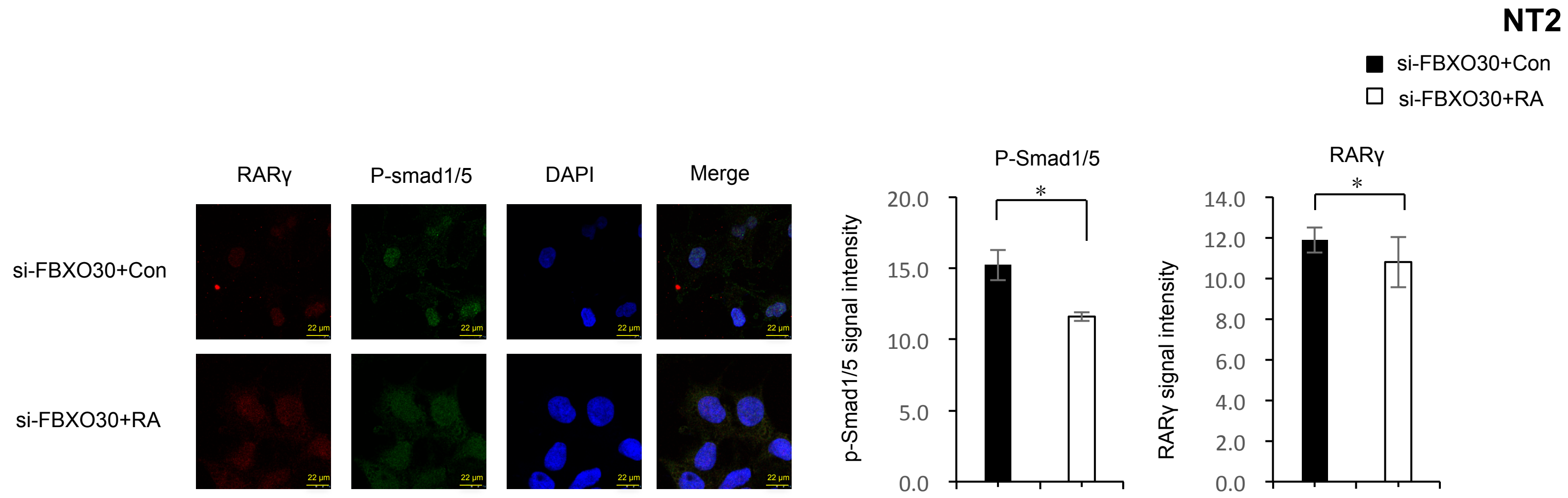

Supp Figure 6

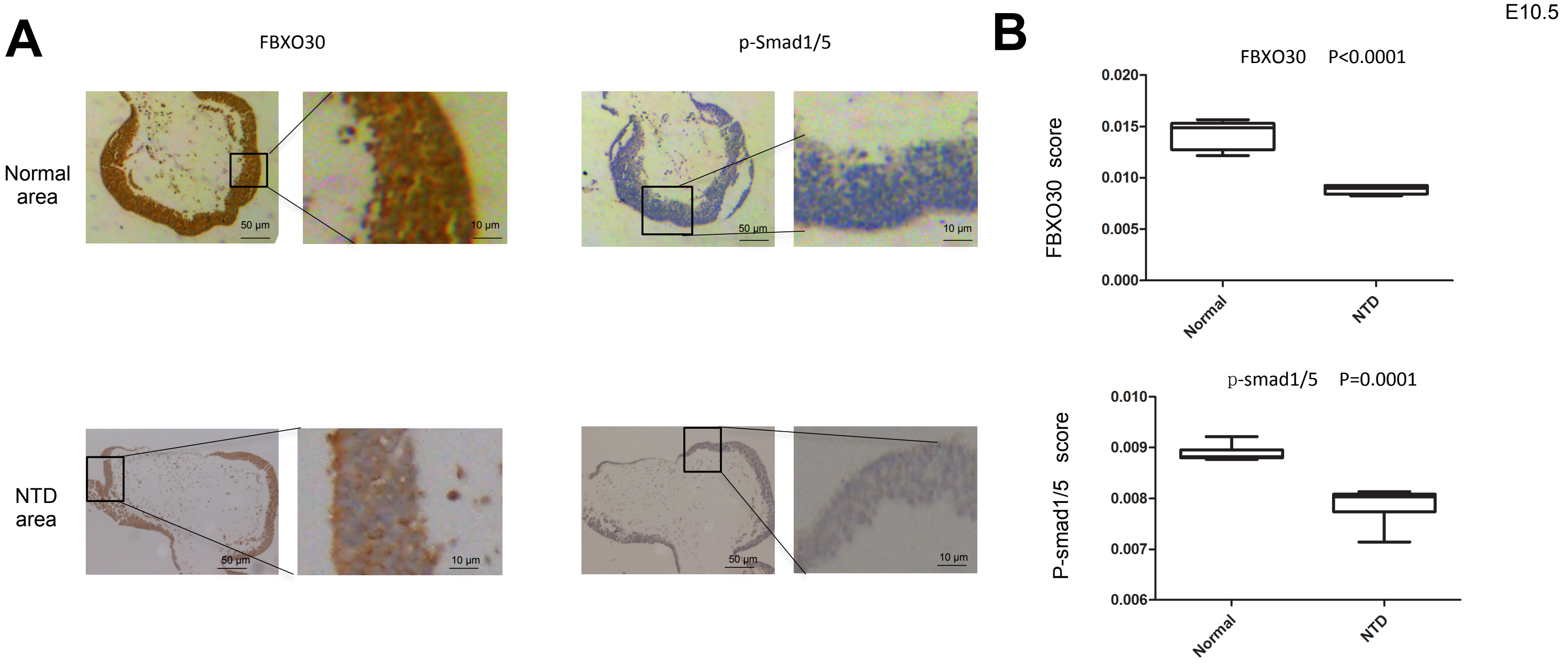

Supp Figure 7

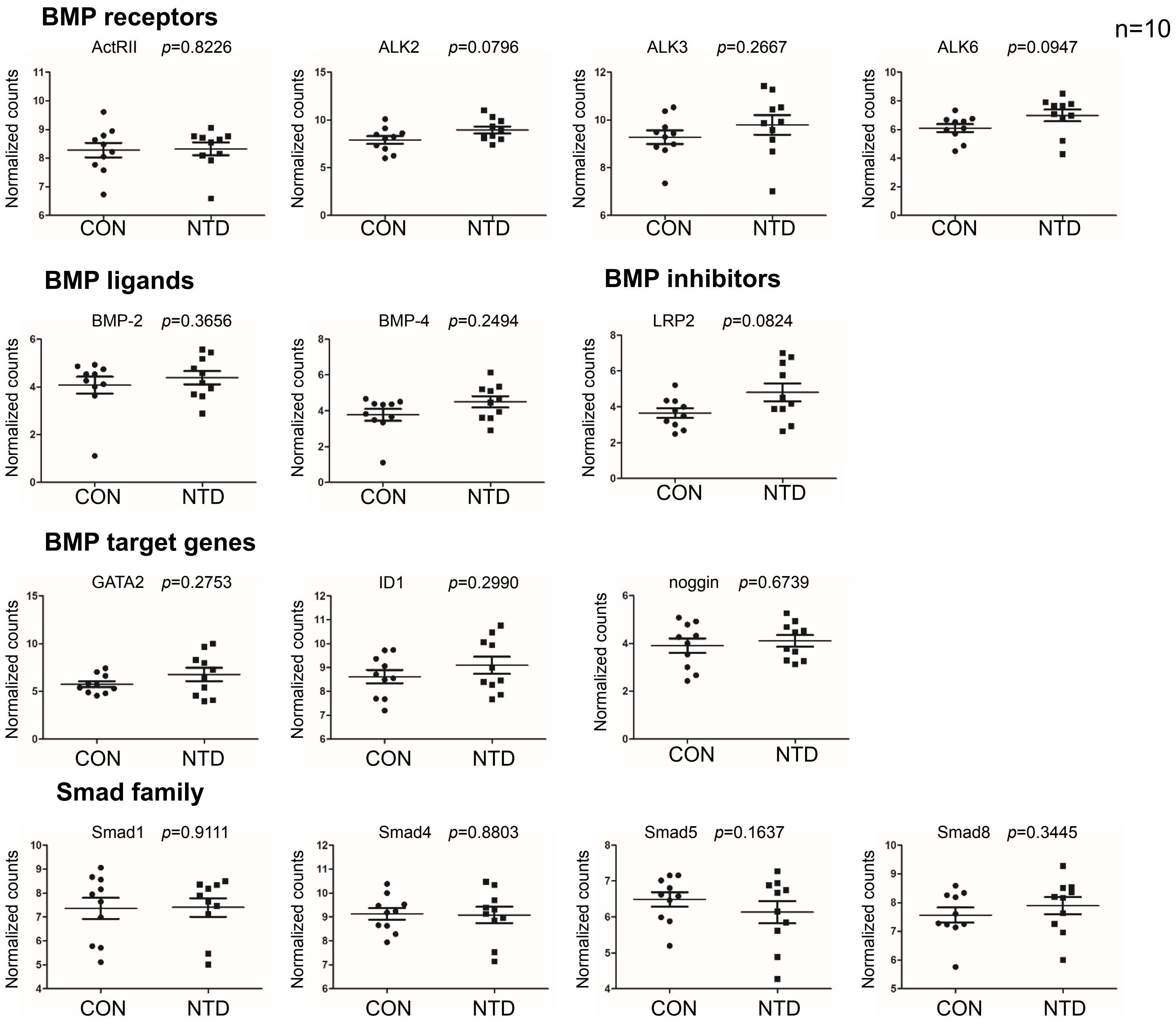

Scan blot Supp Figure 8

Figure 2

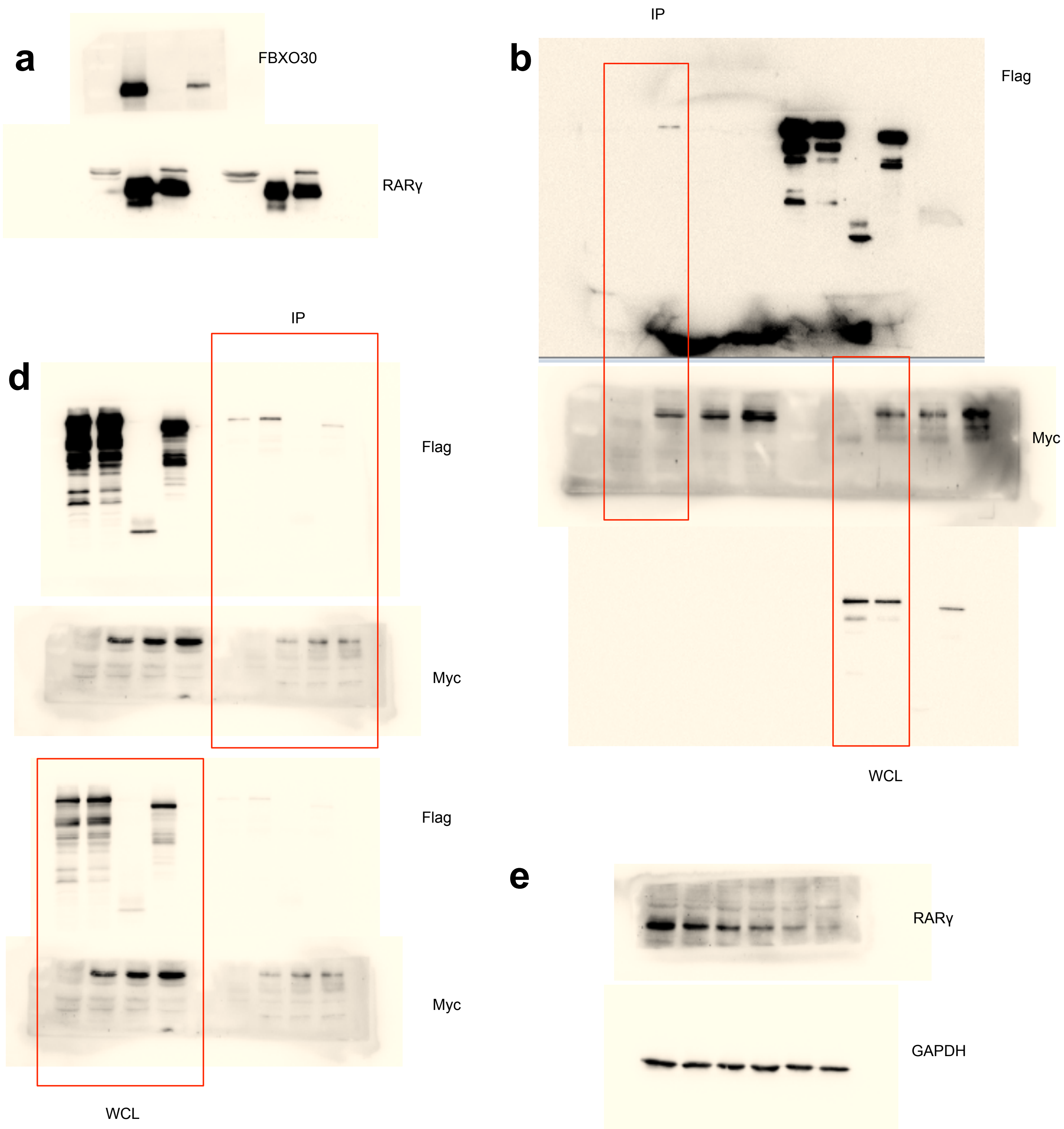

Figure 2

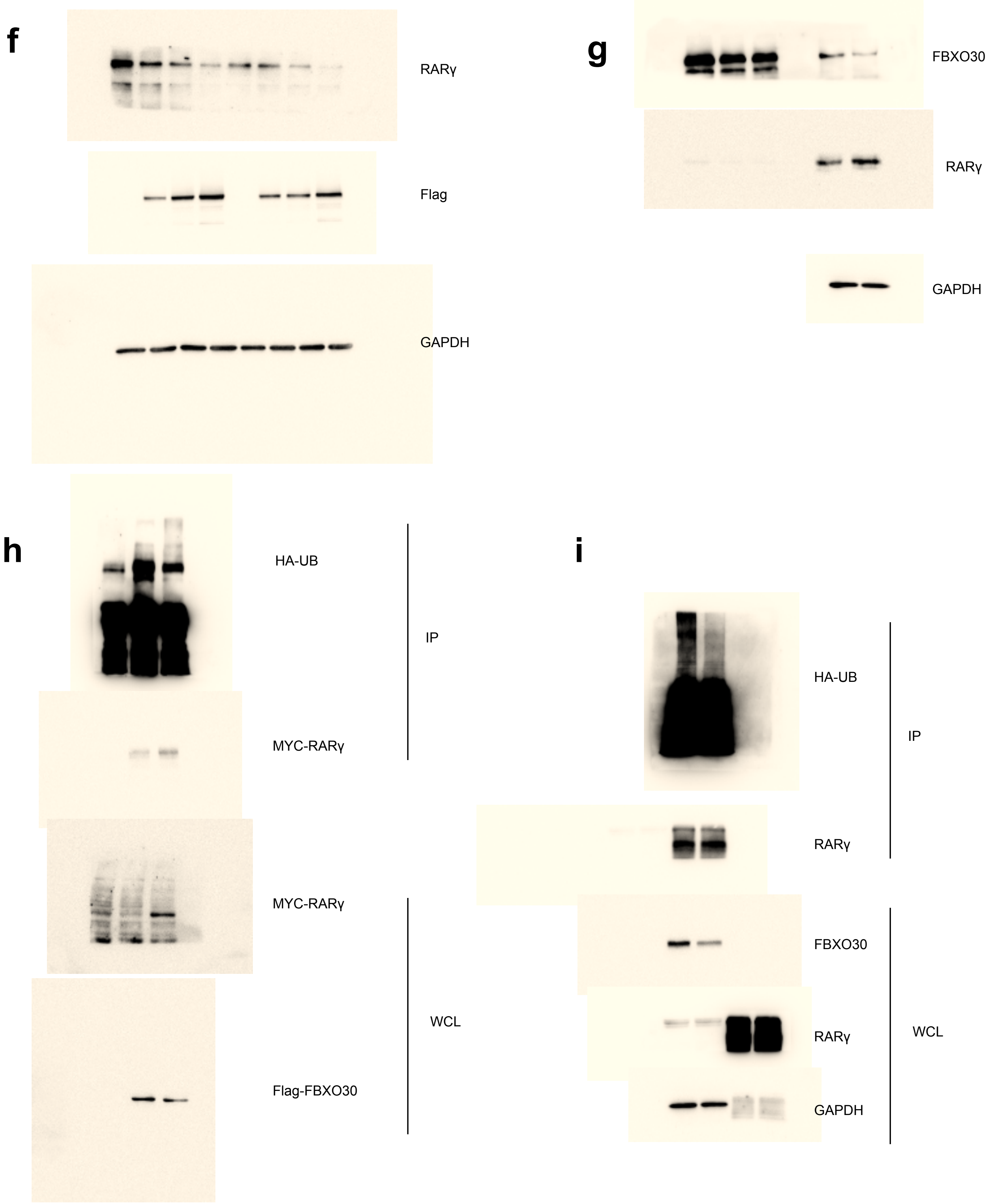
f
RARγ
Flag
GAPDH
g
FBXO30
RARγ
GAPDH
h
HA-UB
MYC-RARγ
IP
MYC-RARγ
WCL
Flag-FBXO30
i
HA-UB
IP
RARγ
FBXO30
RARγ
WCL
GAPDH

Figure 3

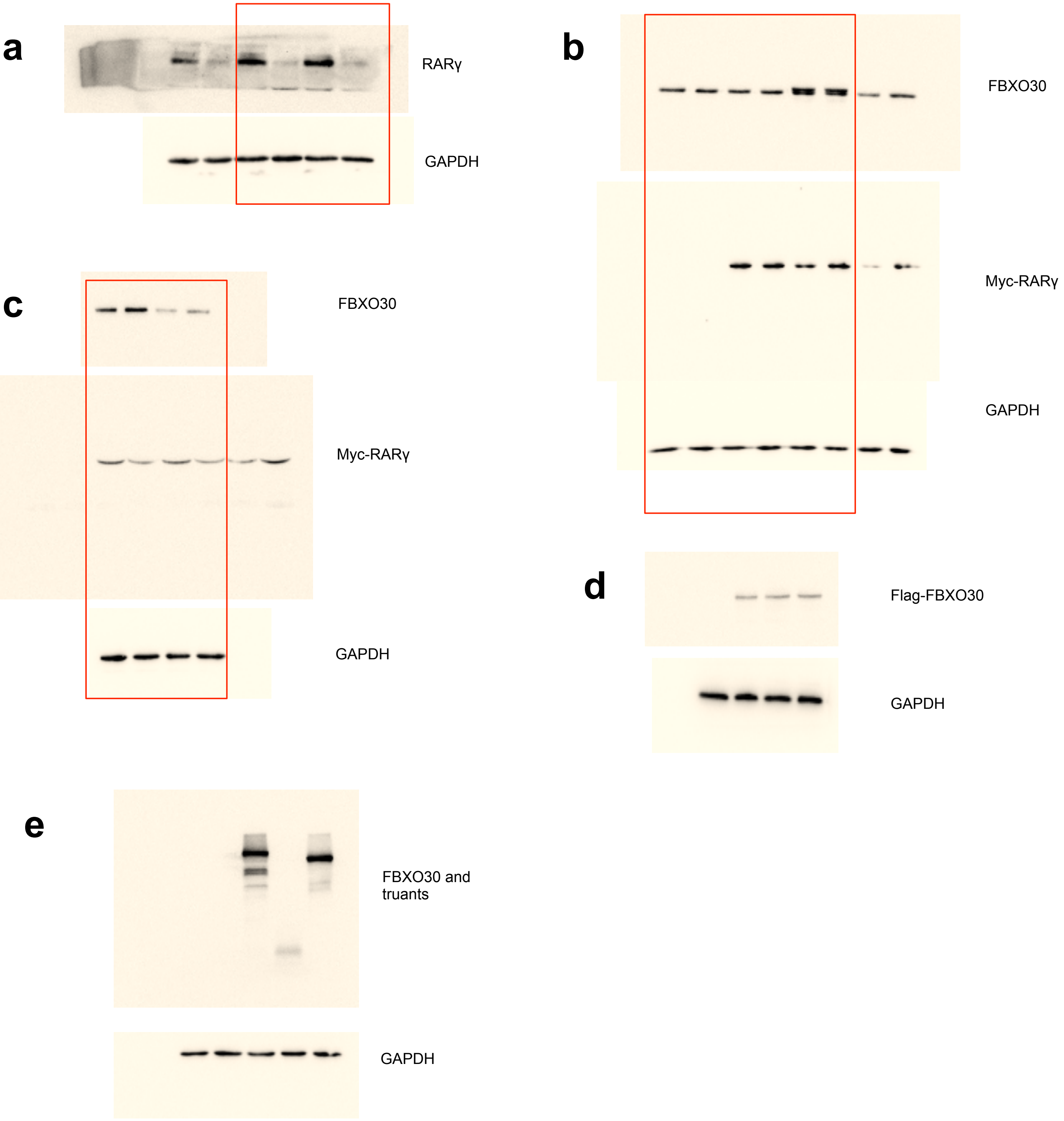

a
RARγ
GAPDH
b
FBXO30
Myc-RARγ
GAPDH
c
FBXO30
Myc-RARγ
GAPDH
d
Flag-FBXO30
GAPDH
e
FBXO30 and
truants
GAPDH

Figure 3

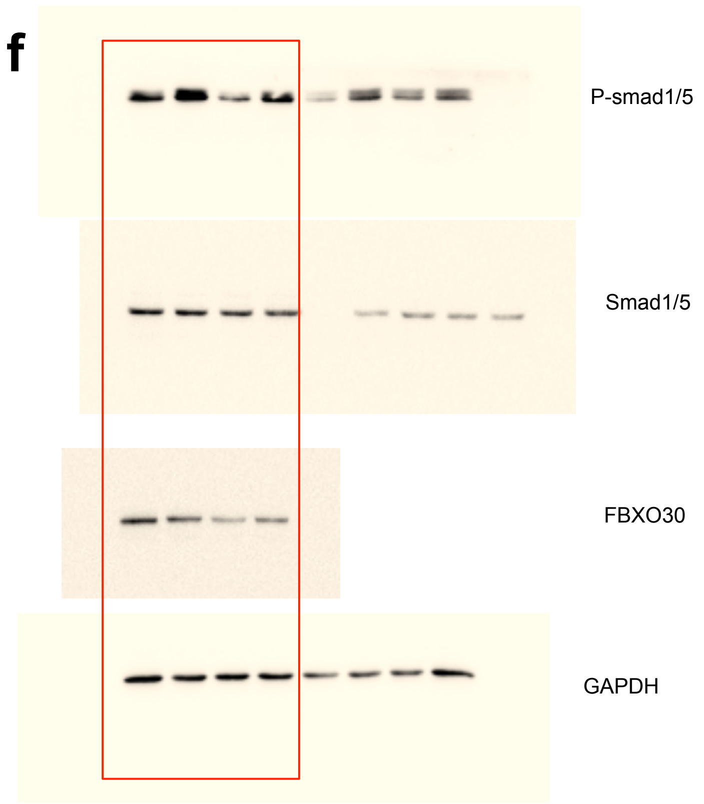
f
P-smad1/5
Smad1/5
FBXO30
GAPDH

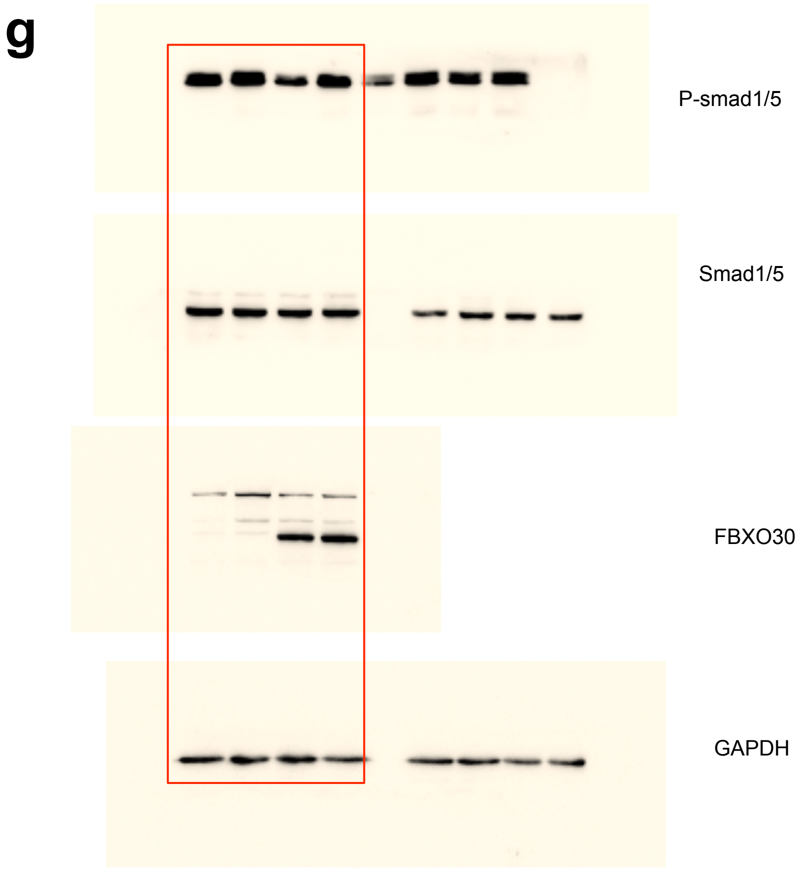
g
P-smad1/5
Smad1/5
FBXO30
GAPDH

Figure 4

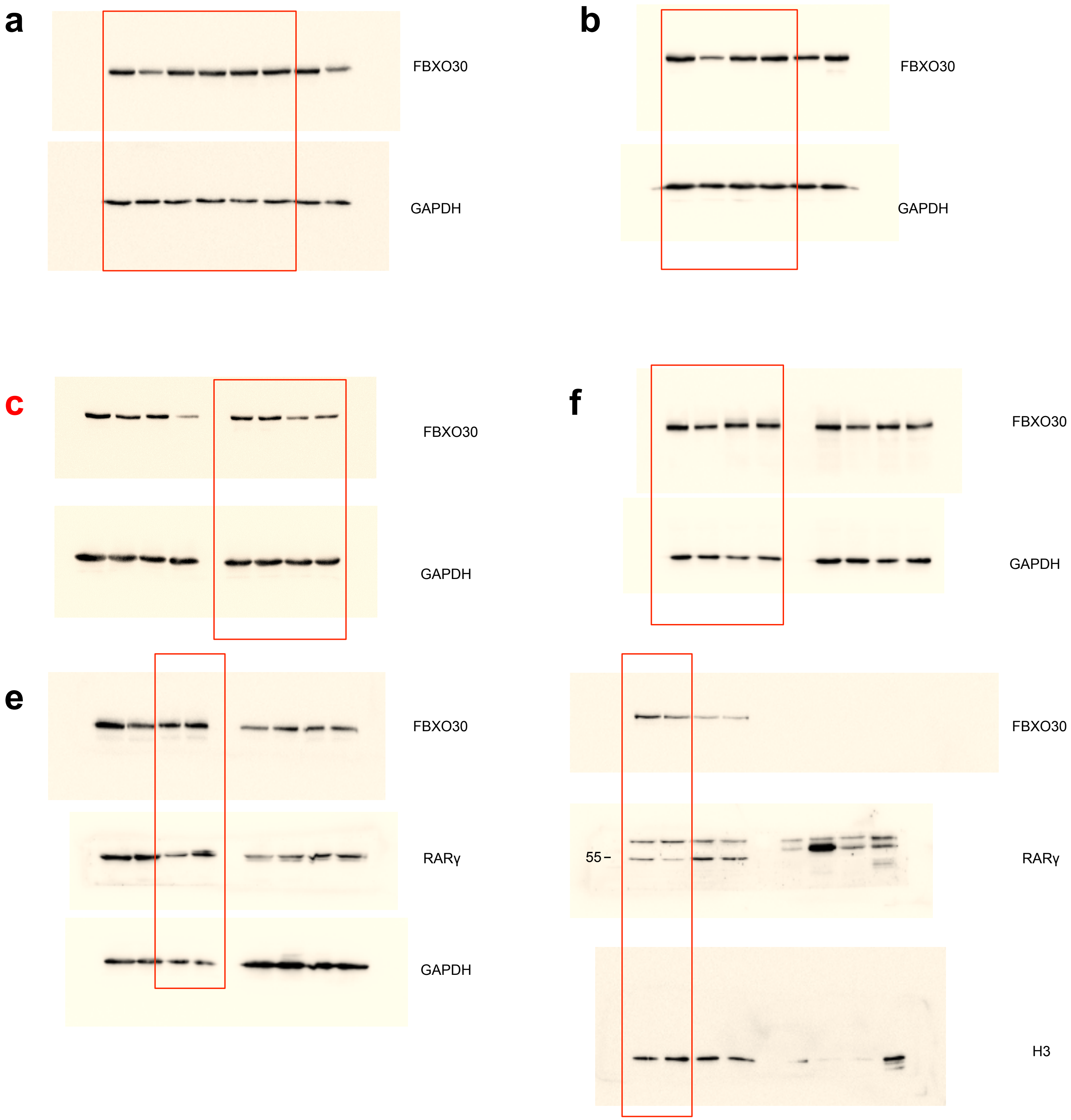

a
FBXO30
GAPDH
b
FBXO30
GAPDH
c
FBXO30
GAPDH
f
FBXO30
GAPDH
e
FBXO30
RARγ
GAPDH
FBXO30
55 –
RARγ
H3

Figure 4

g

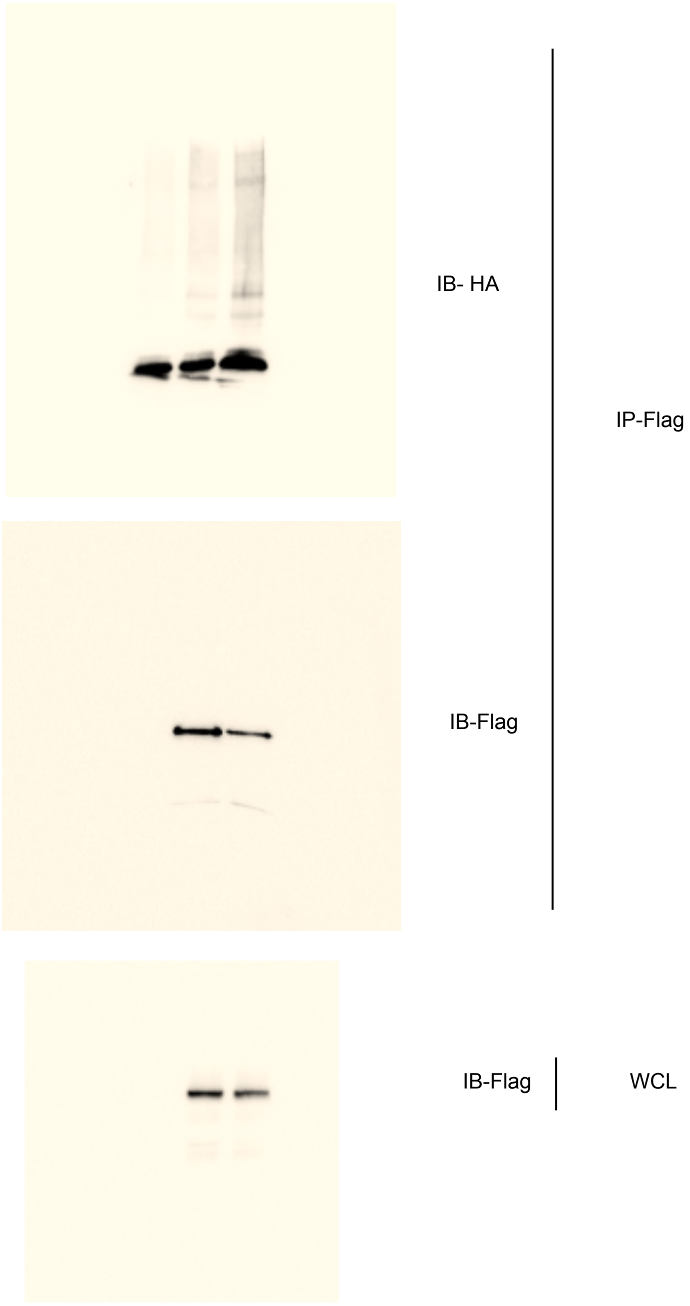

Figure 5

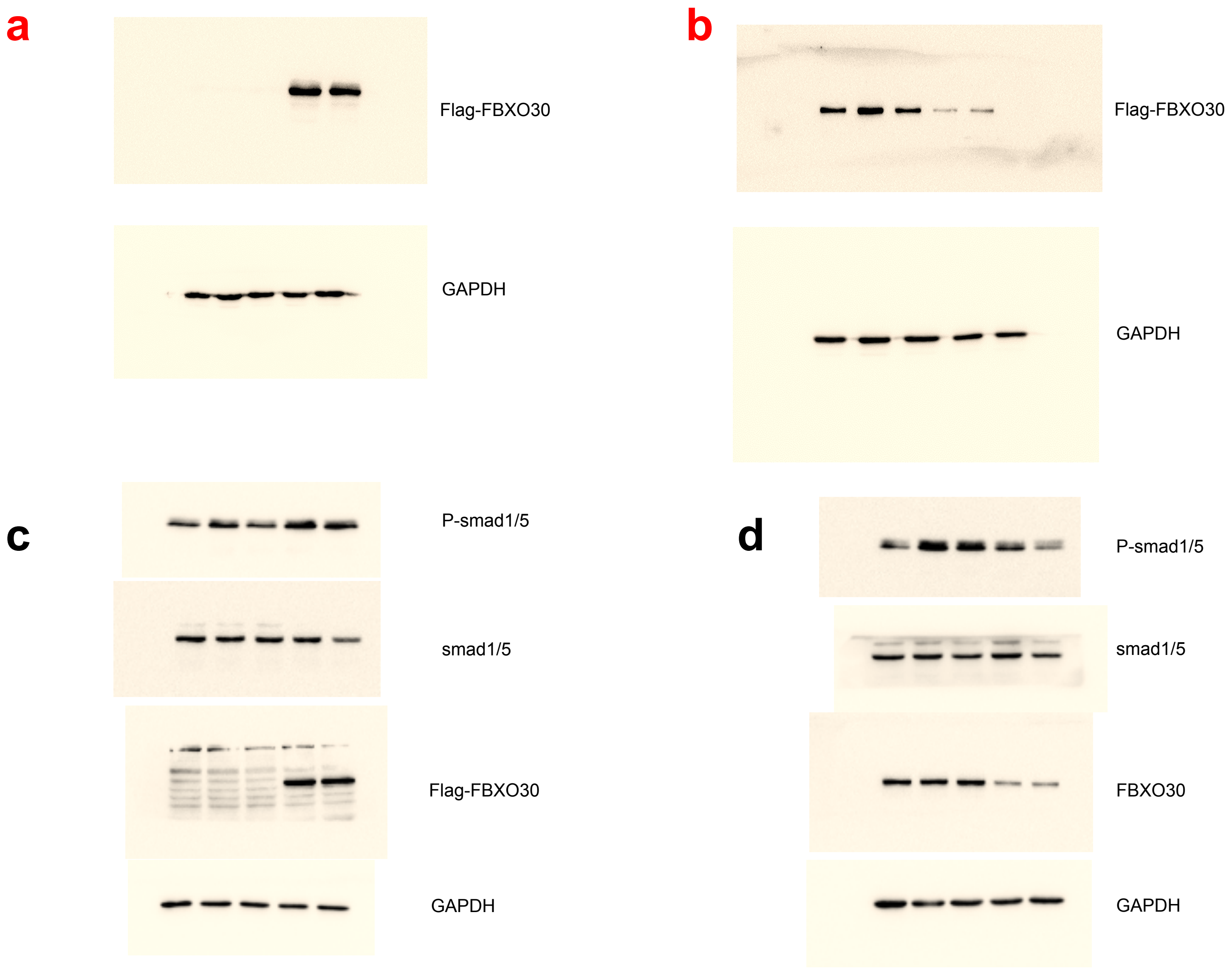
a
Flag-FBXO30
GAPDH
b
Flag-FBXO30
GAPDH
c
P-smad1/5
smad1/5
Flag-FBXO30
GAPDH
d
P-smad1/5
smad1/5
FBXO30
GAPDH

Figure 6

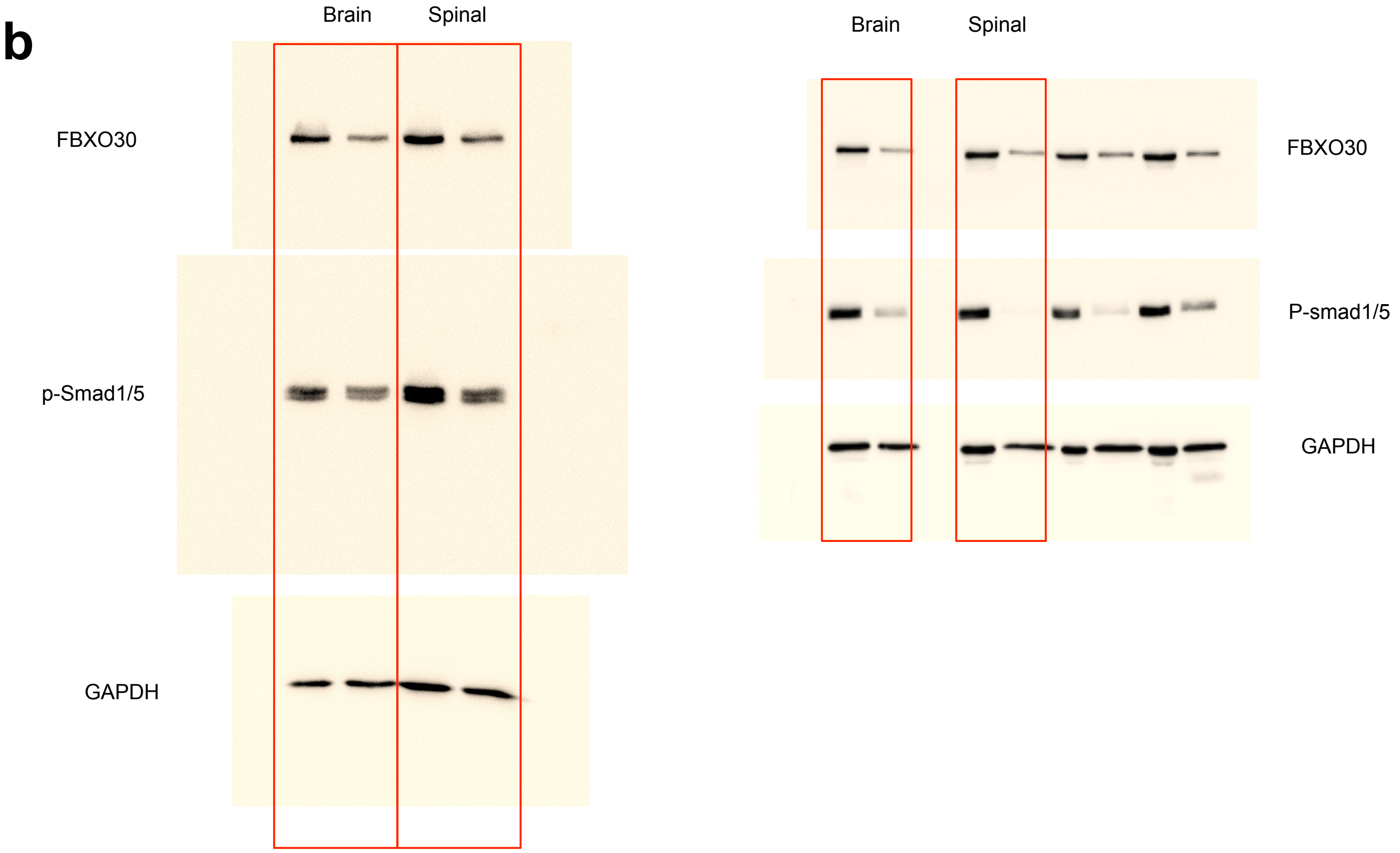
b
Brain
Spinal
FBXO30
p-Smad1/5
GAPDH
Brain
Spinal
FBXO30
P-smad1/5
GAPDH

Figure 7

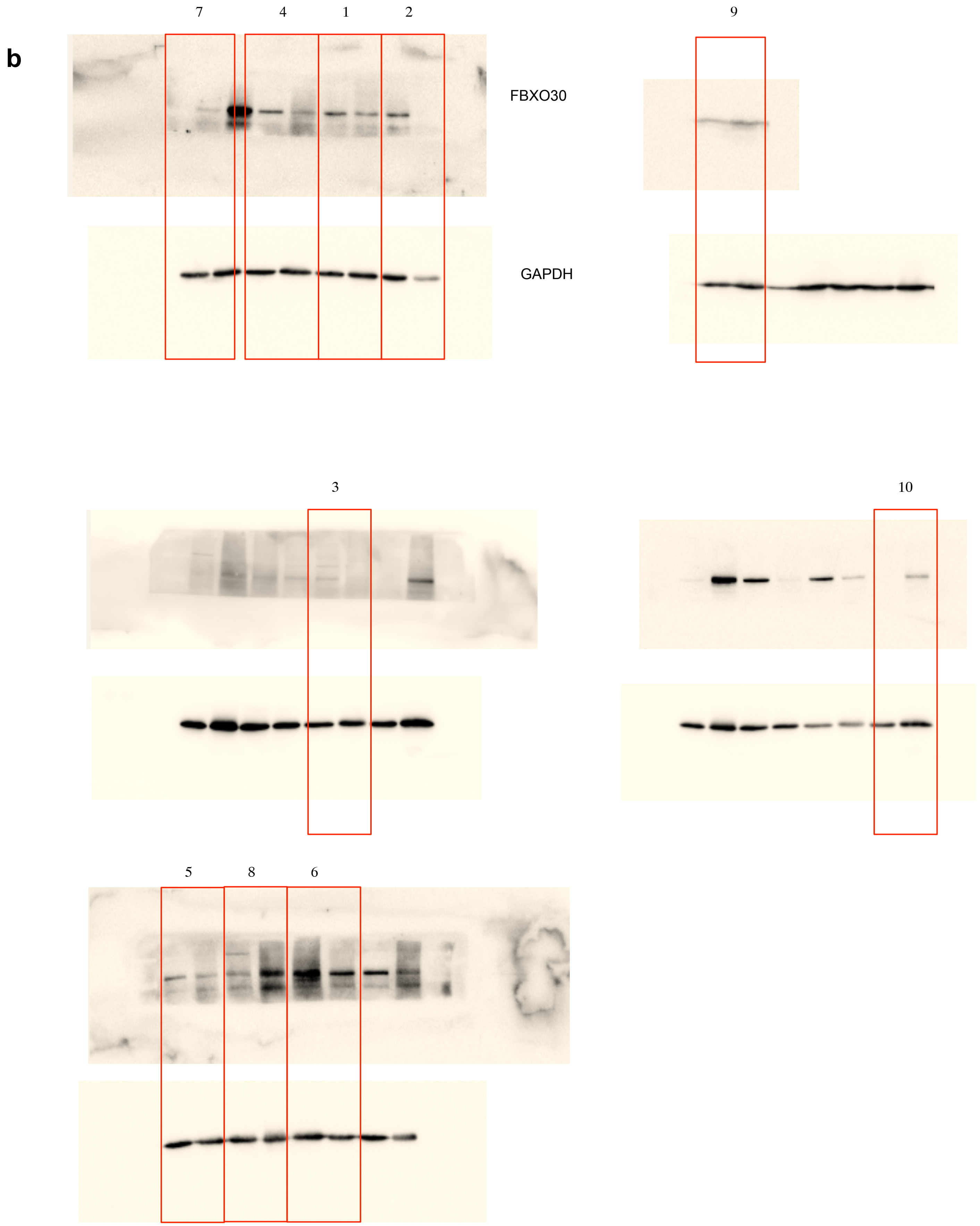

Supp Figure 2

d

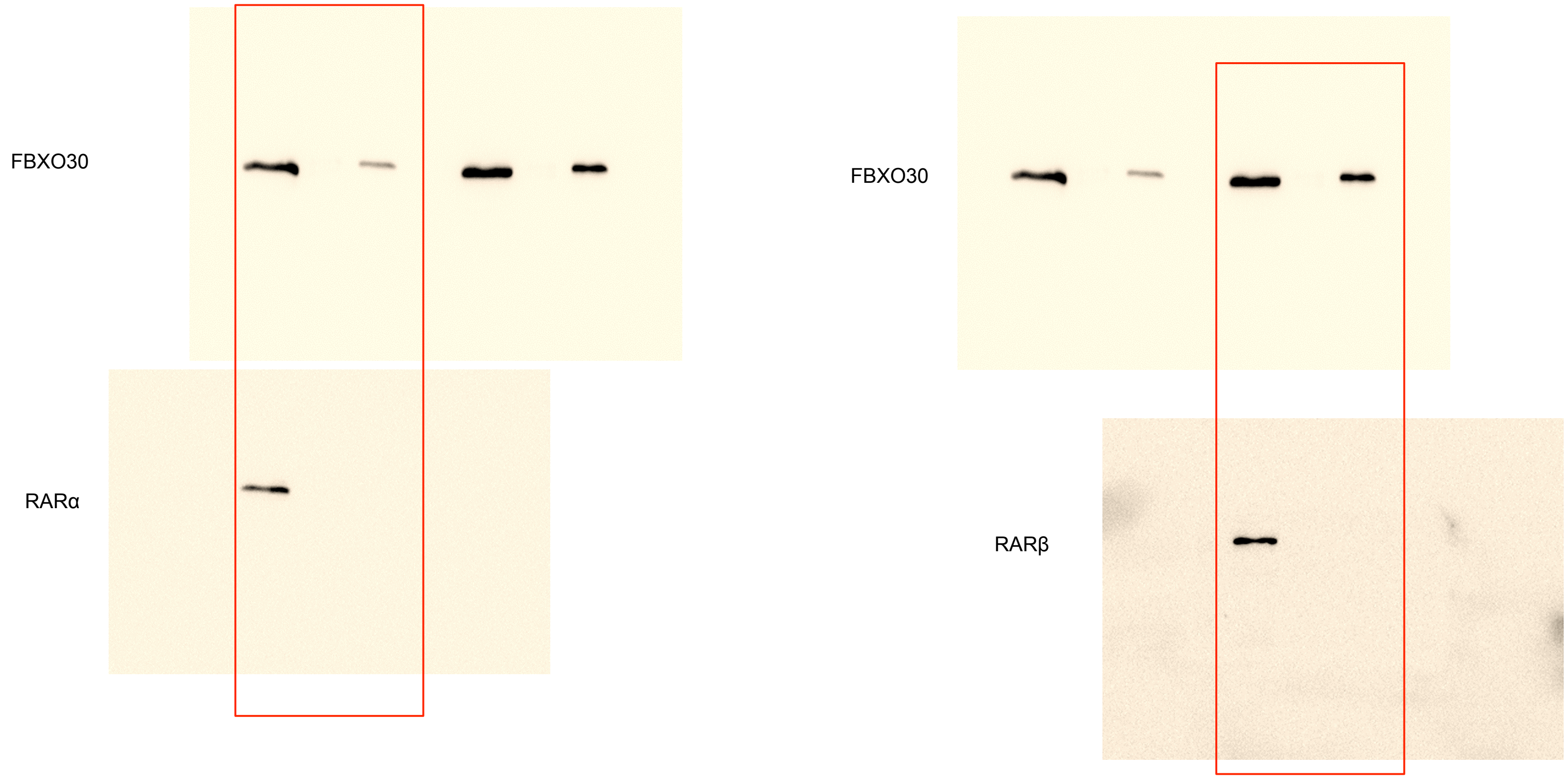

f

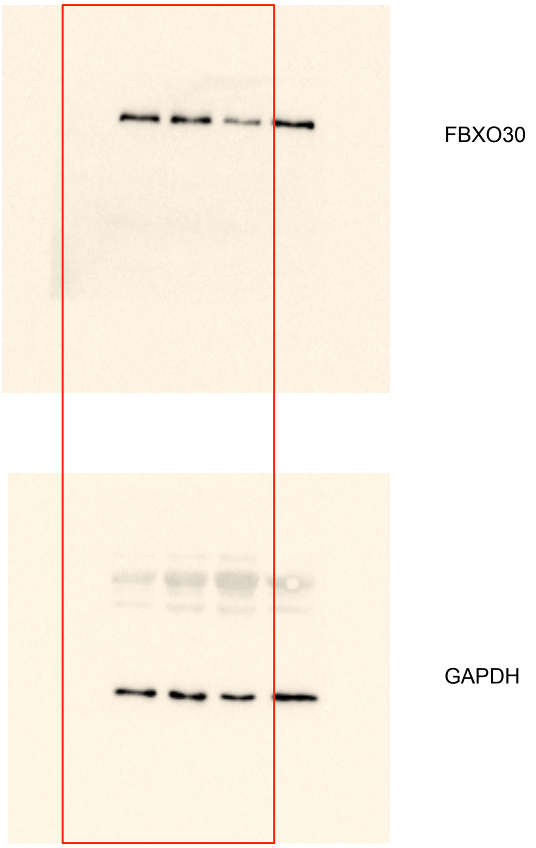

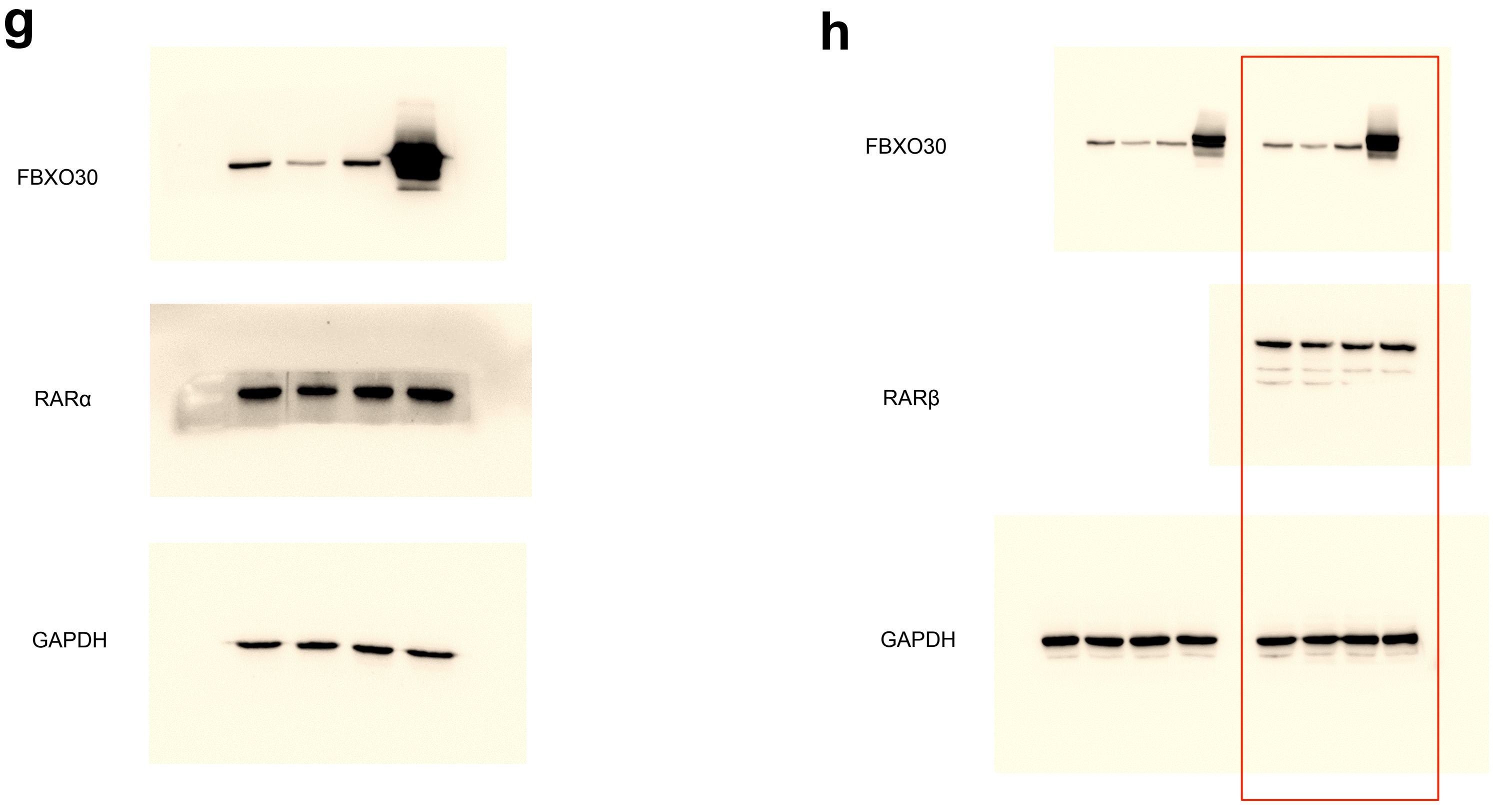

Supp Figure 3

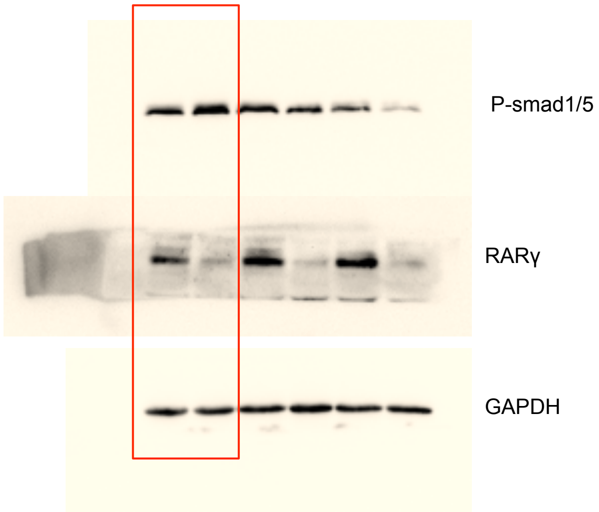

Supp Figure 4

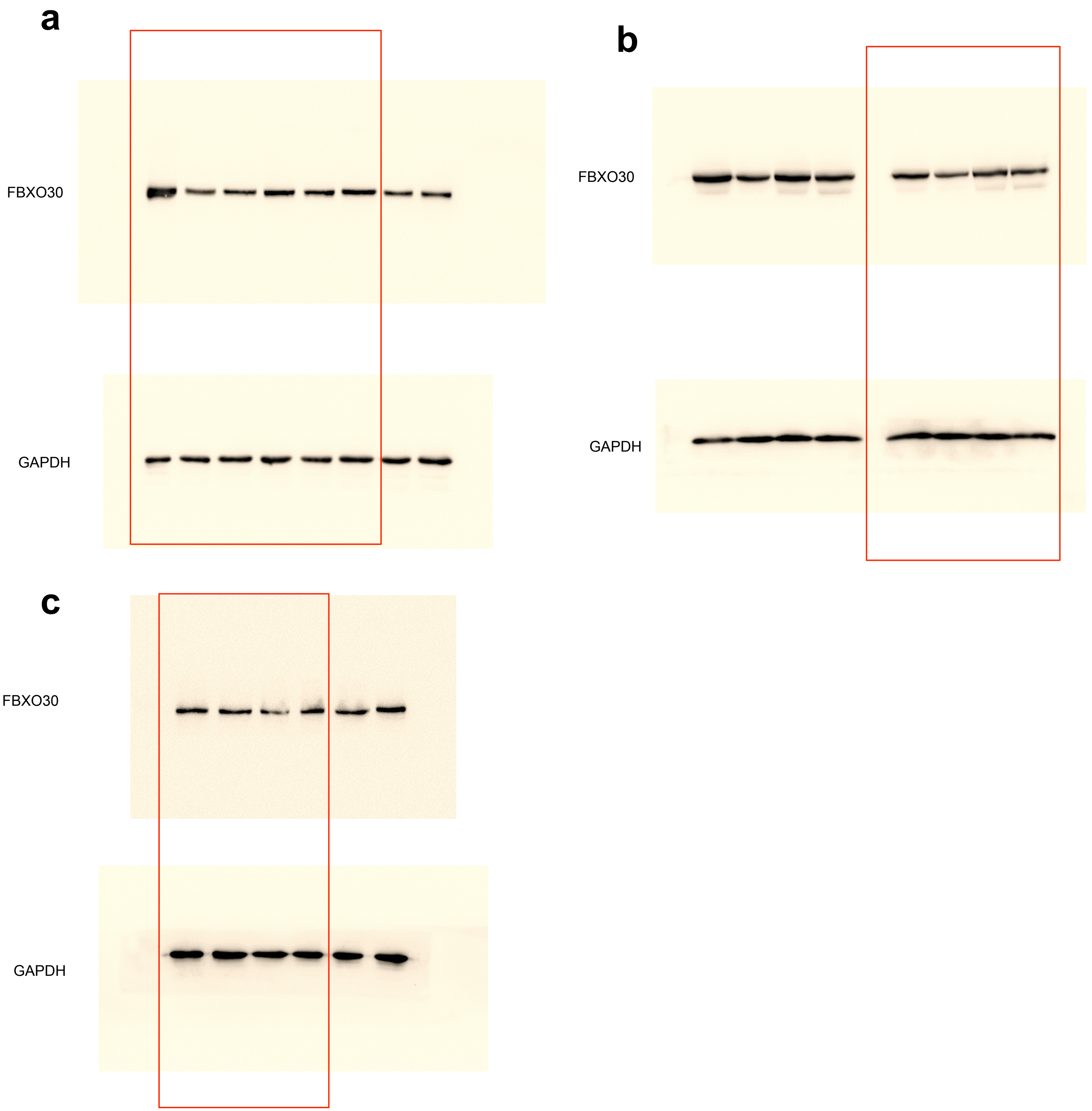

Supp Figure 5

a

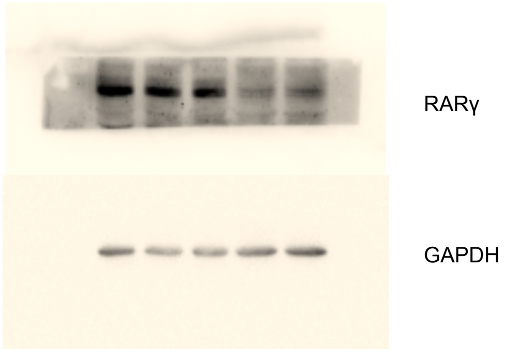

Supplement: Supplementary file 1 — Supp Figures [file 41419_2019_1783_MOESM1_ESM.pdf]
