# Supplementary material for: F-box protein FBXO30 mediates retinoic acid receptor γ ubiquitination and regulates BMP signaling in neural tube defects
Source: Cell Death Dis. 2019 Jul 18;10(8):551. doi: 10.1038/s41419-019-1783-y (PMC6639381; doi:10.1038/s41419-019-1783-y)
Supplement: Supplementary file 2 — Supp informations [file 41419_2019_1783_MOESM2_ESM.pdf]

## Supporting information

### S1 Fig

**A** SiRNA-NC vs siRNA-FBXO30 in NT2/D1 cells. Gene Ontology (GO) enrichmentanalysis for cell component and molecular function of up-regulated proteins. The numbers represent the number of associated proteins for each term. **B** SiRNA-NC vs siRNA-FBXO30 in NT2/D1 cells. Pathway analysis of the DEGs in indicated groups. **C** HEK293 cells were transfected with siRNA FBXO30. Expression of BMP target genes was analyzed by qPCR. All the above data are mean±SD. (n=3), **p*<0.05,by Student's t-test. **D** HEK293 cells were transfected with overexpression of FBXO30.Expression of BMP target genes was analyzed by qPCR. All the above data are mean±s.d. (n=3), **p*<0.05,by Student's t-test

### S2 Fig

**A-C** Mass spectrometry of RARγ interacting proteins in HEK293 cells. The interactions between RARγ and FBXO30 were shown to indicate the dynamic alterations. **D** The whole HEK293 cell extract were immunoprecipitated with anti-RARα or anti-RARβ antibody and Normal rabbit IgG antibody, and analyzed by western blot with anti-FBXO30 and anti-RARα or anti-RARβ. **E** The colocalization of FBXO30 and RARγ in the NT2/D1 cells. Direct immunofluorescence analysis was performed. Images were captured by confocal microscope and the nuclei were stained with DAPI. Scale bars, 22μm. **F** FBXO30 siRNA or NC (negative control) were used to infect HEK293 cells. Knockdown of FBXO30 was detected by immunoblotting and RT–PCR. **G** Overexpression of FBXO30 or knockdown of endogenous FBXO30 in HEK293 cells. Endogenous FBXO30 and RARα or RARβ expression was analyzed by western blot

**S3 Fig**

**A** Knock down endogenous RARγ in HEK293 cells,the expression of p-Smad1/5 was detected by westtern blotting. **B** siRNA-FBXO30 or siRNA-control was transfected into NT2/D1 cells. Direct visualization or indirect immunofluorescence was performed. Scale bars, 22μm.

**S4 Fig**

**A** NT2/D1 cells were treated with RA (1 μM, 12h) or Cispaltin (100μmol/L) or MTX (1μM) for the indicated times. Cell lysates were harvested to detect FBXO30 level (left). Real-time PCR analysis showed no effect of RA on FBXO30 mRNA level (right). Mean values and SD are depicted. **B** NT2/D1 cells were treated with RA 12h for the indicated concentration. Cell lysates were harvested to detect FBXO30 level. **C** NT2/D1 cells were treated with RA (1 μM) for the indicated time. Cell lysates were harvested to detect FBXO30 level. **D** NT2/D1 cells were treated with RA (1 μM) for 12h. Direct visualization or indirect immunofluorescence was performed.

**S5 Fig**

**A** HEK293 cells were transfected siRNA RARγ. Thirty-six hours after transfection, BRE luciferase activity was measured after treatments with BMP-2 (100 ng/ml) and/or RA (1μM) for 12 h. Data are mean±s.d. (n=3). **B** siRNA-FBXO30 or siRNA-normal transfected in NT2/D1 cells as indicated. Thirty-six hours after transfection, cells were stimulated with BMP-2 and/or RA for 12 h before harvesting. Direct visualization or indirect immunofluorescence was performed. Scale bars, 22μm.

**S6 Fig**

**A** Representative images from immunohistochemical staining of FBXO30 and pSmad1/5 in the affect area of cranial neural tissue from E10.5. **B** FBXO30 and pSmad1/5 expression scores are shown as box plots, with the horizontal lines representing the median. Data are mean±s.d. (n=3),*p<0.05, by Student's t-test.

**S7 Fig**

The mRNA expression of BMP related Genes in the brain of NTD fetuses, determined by Nano String. Data are mean±s.d. (n=10),*p<0.05, by Student's t-test.
